# Profiling *Fusobacterium* infection at high taxonomic resolution reveals lineage-specific correlations in colorectal cancer

**DOI:** 10.1038/s41467-022-30957-6

**Published:** 2022-06-09

**Authors:** Dexi Bi, Yin Zhu, Yaohui Gao, Hao Li, Xingchen Zhu, Rong Wei, Ruting Xie, Chunmiao Cai, Qing Wei, Huanlong Qin

**Affiliations:** 1 Department of Pathology, Shanghai Tenth People’s Hospital, Tongji University School of Medicine, Shanghai, 200072 China; 2 Department of Gastrointestinal Surgery, Shanghai Tenth People’s Hospital, Tongji University School of Medicine, Shanghai, 200072 China

**Keywords:** Colorectal cancer, Microbiome, Colorectal cancer

## Abstract

The bacterial genus *Fusobacterium* promotes colorectal cancer (CRC) development, but an understanding of its precise composition at the species level in the human gut and the relevant association with CRC is lacking. Herein, we devise a *Fusobacterium rpoB* amplicon sequencing (FrpoB-seq) method that enables the differentiation of *Fusobacterium* species and certain subspecies in the microbiota. By applying this method to clinical tissue and faecal samples from CRC patients, we detect 62 *Fusobacterium* species, including 45 that were previously undescribed. We additionally reveal that *Fusobacterium* species may display different lineage-dependent functions in CRC. Specifically, a lineage (designated L1) including *F. nucleatum*, *F. hwasookii*, *F. periodonticum* and their relatives (rather than any particular species alone) is overabundant in tumour samples and faeces from CRC patients, whereas a non-enriched lineage (designated L5) represented by *F. varium* and *F. ulcerans* in tumours has a positive association with lymphovascular invasion.

## Abstract (web summary)

Bacteria from the genus *Fusobacterium* can promote colorectal cancer (CRC) development; however, the exact *Fusobacterium* species involved in this process remain underexplored. Here, the authors develop a *rpoB* amplicon sequencing approach to identify *Fusobacterium* species and subspecies in CRC patient samples.

## Introduction

Colorectal cancer (CRC) is a common malignant disease worldwide. The gut microbiota is now recognised as an important player in CRC^1^. Emerging evidence has demonstrated that the bacterial genus *Fusobacterium* in the human gut promotes CRC development^2–5^.

Among *Fusobacterium* members, *Fusobacterium nucleatum* is the most studied species that is associated with CRC^3^. It is enriched in the gut microbiota of CRC patients^5^ and promotes carcinogenesis through multiple mechanisms, including increasing tumour cell proliferation by upregulating miR-21^6^, promoting carcinogenesis by modulating E-cadherin/β-catenin signalling and inducing Wnt/β-catenin^7,8^, inducing chemoresistance by modulating autophagy^9^, and protecting tumours from immune cell attack^10^. It can also be found in the liver metastases of *Fusobacterium*-associated primary tumours^11^. Although most CRC-related studies have chosen *F. nucleatum* (especially, subsp. *nucleatum*) as a model organism, the precise compositions of gut *Fusobacterium* communities at the species level remain largely unknown. Yet, a metagenomic study revealed that there were multiple fusobacterial taxa in the gut of southern Chinese populations^12^. A similar study in the same population showed that non-*F. nucleatum* species were also predominant and might have distinctive correlations with different host diseases^13^. Moreover, *F. nucleatum* itself has four subspecies, namely, *nucleatum*, *animalis*, *vincentii* (inclusive of *fusiforme*) and *polymorphum*, which are phylogenetically divergent and can arguably be classified as separate species^14,15^. It has been suggested that subsp. *animalis*—but not subsp. *nucleatum*—might be prevalent in CRC tissues^16^. Therefore, despite the extraordinary progress achieved in determining the oncogenic mechanisms of *F. nucleatum* with type strains, the uncertainties in the compositions of gut *Fusobacterium* communities have complicated efforts to establish aetiological relationships between *Fusobacterium* members and CRC. Understanding the species-level composition of *Fusobacterium* community is important to elucidate the roles played by *Fusobacterium* in the development of CRC and could provide precise targets that may aid early diagnosis and even treatment of CRC.

Major gut microbiota profiling approaches include 16S rRNA gene amplicon sequencing and metagenomic sequencing. But the 16S rRNA gene displays high similarity within *Fusobacterium*, and furthermore, it does not provide species-level resolution for this genus when used in amplicon sequencing^13,17^. In contrast, *rpoB*, which is also a widely used prokaryotic genotyping marker in the assessment of genetic relatedness, is sufficiently polymorphic to distinguish *Fusobacterium* species^17^. The metagenomic sequencing is capable of species identification, but it relies mostly on reference genomes of known species.

This study aims to resolve the precise compositions of gut *Fusobacterium* communities in CRC patients to probe the roles played by *Fusobacterium* members. By taking advantage of comparative genomic analysis, we devise a method that allows *Fusobacterium* species and certain subspecies to be differentiated in the microbiota and investigated the compositional features of *Fusobacterium* communities in the tumours and faeces of CRC patients. This study may promote the understanding of how *Fusobacterium* members promote CRC development and provide further directions to better model this malignant disease and study relevant host-gut microbe interactions.

## Results

### Species-level taxonomic analysis of *Fusobacterium* with sequenced genomes

**Fig. 1:**
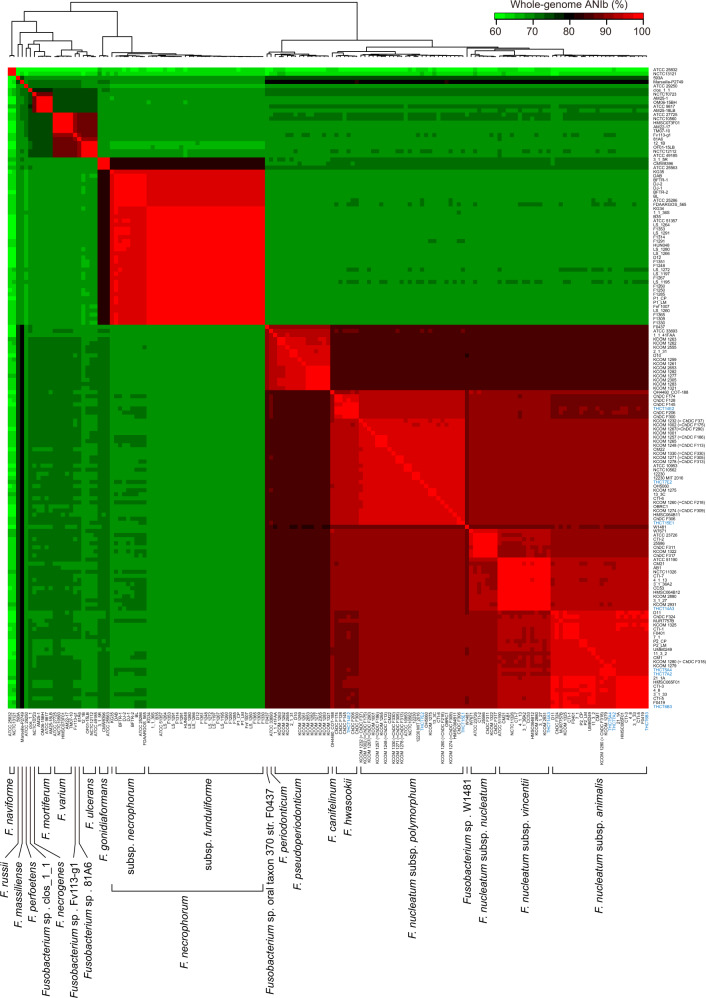
Whole-genome ANIb analysis effectively defined *Fusobacterium* species and reclassified mislabelled species annotations for genomes in the NCBI database. A total of 157 *Fusobacterium* genomes were used for pairwise ANIb analysis. A 157 × 157 matrix of ANIb values (Supplementary Data 2) calculated for all strains in a pairwise manner is presented as a heatmap. Strain names are listed on the right side of and under the heatmap. Blue names denote strains sequenced in this study. The resulting species/subspecies designation is shown at the bottom. ANIb, average nucleotide identity calculated with BLAST.

To develop a new approach that can differentiate *Fusobacterium* species and some subspecies in the microbiota, we sought to search for taxonomic marker candidates in sequenced *Fusobacterium* genomes. A total of 150 genomes from the NCBI database and seven genomes sequenced by this study (Supplementary Data 1) were used. Taxonomic analysis of the *Fusobacterium* genus was firstly conducted with the genomes to serve as a reference for subsequent investigation. Whole-genome ANIb analysis was performed in a pairwise manner to ensure accurate taxonomic designation (Supplementary Data 2 and Fig. 1), as there could be inaccurately labelled species names in the database. The species names of 17 strains were reassigned, while 15 *F. nucleatum* and 10 *Fusobacterium necrophorum* strains were classified further into subspecies (Supplementary Data 1). We found that similar to most bacteria^18^, an ANIb cut-off of 94% could effectively delineate *Fusobacterium* species (Fig. S1), except *F. nucleatum*. The *F. nucleatum* strains commonly displayed >90% ANIbs with each other, but 90% ANIb could not be used as the species boundary since the strains also showed >90% ANIbs with *F. hwasookii* and *Fusobacterium canifelinum*. However, the 94% ANIb accurately defined the four *F. nucleatum* subspecies (*nucleatum*, *animalis*, *polymorphum* and *vincentii*), suggesting that these subspecies can be considered as four separate species, consistent with previous reports^14,15^. We also found that the *F. necrophorum* strains could be categorised into three clades, two of which corresponded to the subspecies *necrophorum* and *funduliforme*, while one might represent a different subspecies. However, unlike *F. nucleatum*, all members of *F. necrophorum* showed >95% intra-species ANIbs, and the subspecies boundaries were apparent at a cut-off of 97.5%. The 157 strains were ultimately classified into 19 species and four *F. nucleatum* subspecies (Fig. 1). The classification was in accordance with the whole-genome phylogeny of these genomes (Fig. S2). Of note, in the whole-genome phylogenetic tree, the *F. nucleatum* subspecies were also not in the same clade but were instead located on distinct major branches parallel to other species, which again supported the hypothesis that the four subspecies should rank as species. Thus, in the following analyses, they were considered as separate species (unless otherwise specified). As such, we clarified the taxonomy of *Fusobacterium* based on whole-genome analyses.

### Development of a *Fusobacterium rpoB* amplicon-sequencing (FrpoB-seq) method for species-level designation of *Fusobacterium*

**Fig. 2:**
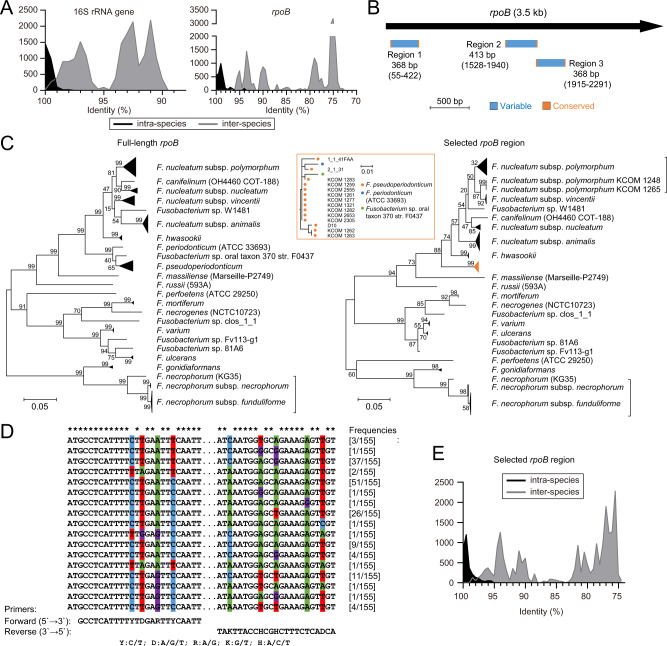
Development of a *rpoB*-based approach for *Fusobacterium* differentiation at the species level. **A** Distributions of pairwise intra- and inter-species identities of the 16S rRNA (*n* = 2610 and *n* = 17,696, respectively) and *rpoB* genes (*n* = 3068 and *n* = 20,802, respectively) from the genomes. **B** Locations of three candidate regions compatible with amplicon sequencing found in *rpoB*. The reference length and coordinates (in parentheses) in the *rpoB* gene of *F. nucleatum* subsp. *nucleatum* ATCC 25586 are given. Region 1 was selected for further study. **C** Phylogenetic trees of full-length *rpoB* and their corresponding selected *rpoB* region. Strain names are given in parentheses for the species with only one sequenced genome available. Branches of the same species/subspecies or those otherwise illustrated in the orange box (corresponding to the orange triangle) are compressed as applicable. There is an exception in the sub-tree of *F. necrophorum* subsp. *funduliforme*, which is illustrated in Fig. S3. Strain names are also provided for those that could not be compressed together. **D** Alignment of the non-redundant terminal conserved sequences of the selected *rpoB* region and universal primers designed based on these conserved regions. Asterisks denote consensus bases. Variations are denoted by nucleotide-specific colour shades. **E** Distribution of pairwise intra- and inter-species identities of the selected *rpoB* region (*n* = 3068 and *n* = 20,802, respectively). Complete gene sequences available in the sequenced genomes were used for analysis (144 16S rRNA gene and 155 *rpoB* sequences). *F. naviforme* genomes (*n* = 2) were not included. The analysis considered the four *F. nucleatum* subspecies as separate species. Source Data are provided as a source data file.

Based on the genomic data, we found that *rpoB* was an accurate high-resolution taxonomic marker for *Fusobacterium*. The *rpoB* was present in one copy in each genome. The phylogenetic trees based on *rpoB* and the 16S rRNA gene were both capable of distinguishing *Fusobacterium* species, showing consistency with the taxonomy described above (Fig. S3). Moreover, *rpoB* was more polymorphic than the 16S rRNA gene for species-level identification, as its intra- and inter-species identities had almost separable distributions (Fig. 2A). In the *rpoB* tree, all the studied species formed distinct clades, outperforming the 16S rRNA gene tree (Fig. S3). In particular, *rpoB* could clearly distinguish *Fusobacterium periodonticum* and *Fusobacterium pseudoperiodonticum* and even the subspecies of *F. necrophorum*, with only one exception, namely, that an *F. necrophorum* subsp. *necrophorum* strain (DAB) was grouped in the clade of *F. necrophorum* subsp. *funduliforme* (Fig. S3). We also noted that in both the *rpoB* and 16S rRNA gene trees, the four *F. nucleatum* “subspecies” formed separate clades parallel to other species, in line with the whole genome-based findings (Fig. S3). Notably, all the studied *Fusobacterium* species could be robustly classified into certain lineages (Fig. S3). Thus, the *rpoB* was employed to develop a *Fusobacterium* species differentiation method.

We next performed multiple sequence alignment of the *Fusobacterium rpoB* genes to search for a short representative region compatible with amplicon sequencing. We searched for polymorphic regions of 300–500 bp bounded by >20 bp conserved sequences. Three candidate regions were identified (Fig. 2B), and only Region 1 was selected as it was found to be specific to *Fusobacterium* based on comparison against the NCBI nucleotide database. The selected region was ~370 bp in length and located close to the 5′ end of the gene. Notably, the phylogeny of this region retained the capacity of distinguishing *Fusobacterium* species (Fig. 2C). The termini of the region were ~25 bp in length and highly conserved among *Fusobacterium* species, based on which a set of universal primers was designed (Fig. 2D). The primers yielded a single specific band from different *Fusobacterium* isolates (Table S1) and human faecal samples (Fig. S4A). We also assessed whether the primers were specific to *Fusobacterium* using in silico analysis against the NCBI database. Certain putative non-specific amplification was detected with multiple primer mismatches, but most of the species detected were environmental or animal-inhabiting microorganisms (Table S2). Therefore, this region was adopted for *Fusobacterium rpoB* amplicon sequencing (FrpoB-seq). Based on ANIb analysis, we were unable to define a species boundary of sequence identity for this region, as it had 95.1–100% (median: 99.7%) intra-species and 74.7–99.7% (median: 80.4%) inter-species identities (Fig. 2E), we, therefore, adopted a phylogeny-based annotation strategy in the following analysis. But in most cases, 98% could be considered as an efficient boundary (Fig. 2E) and was also used to aid annotation. Notably, *Fusobacterium naviforme* was not included in the analysis, as its *rpoB* gene did not show such conservation. Moreover, *F. naviforme* showed a distant relationship to other studied *Fusobacterium* species (Fig. 1 and S3). Finally, we set up mock samples and tested the sensitivity of FrpoB-seq. We found the limit to achieve successful construction of a standard sequencing library was 1000–10000 overall *Fusobacterium rpoB* copies per reaction, while upon successful FrpoB-seq, detection of a particular species could be achieved at ~100 *rpoB* copies per reaction. In addition, the detection result was generally in accord with the composition in mock samples (Fig. S4B). With the above efforts, we developed a FrpoB-seq method for species-level designation of *Fusobacterium*.

### Implementation of FrpoB-seq in tissue and faecal samples of CRC patients

FrpoB-seq was then conducted with tissue (551 tumour-adjacent tissue pairs) and faecal samples from CRC patients (*n* = 94) and healthy individuals (*n* = 95). FrpoB-seq was accomplished in 556 (304 tumour and 252 normal) tissue and 99 (61 CRC and 38 control) faecal samples. Factors resulting in unaccomplished implementation included low or non-detectable *Fusobacterium* abundance, low amounts of sample DNA, and/or limited library quality. Notably, most of the sequencing data (91.42% and 99.73% in tissue and faecal samples, respectively) were specific to the *Fusobacterium rpoB* target (Fig. S4C). Non-specific data mainly corresponded to the DNA of humans and some gut microbes (Table S3). The study used a 100% threshold to generate operational taxonomic units (OTUs), which were subsequently subjected to phylogeny-based species annotation (Fig. S5). Strikingly, 62 species were identified, and 45 (72.6%) of them were previously undescribed (Supplementary Data 3).

The *rpoB* sequences of the previously undescribed species all showed <98% (mostly <97%) identity to those of the known species, also sufficient to classify them as separate species. To further validate those species, the study performed deep metagenomic sequencing (average ~10 Gbp) in a subset (*n* = 35; Supplementary Data 3) of faecal samples covering 25 putative undescribed species (Fig. S6). The existence of five putative undescribed species was directly confirmed in the matching samples (Fig. S6). However, in samples associated with the remaining putative undescribed species, the selected *rpoB* region or even the entire *rpoB* gene was not covered by metagenomic sequencing, likely due to the low abundance of *Fusobacterium* communities. We alternatively search for *Fusobacterium*-specific *rpoB* fragments of other region, 16S rRNA genes and other genomic sequences that belonged to undescribed species and indeed the corresponding evidence was obtained (Fig. S6). But in such cases, the FrpoB-seq data could not be exactly mapped. We additionally searched for the *rpoB* of undescribed *Fusobacterium* species in the public metagenomic datasets of two cohorts, a Southern Chinses population cohort (*n* = 556, average ~7.5 Gbp) reported containing non-*nucleatum Fusobacterium*^12^, and a Korean population cohort (*n* = 106, average >30 Gbp) with ultra-high sequencing depth^19^. Eight putative undescribed *Fusobacterium* identified by this study were confirmed in the two cohorts (Fig. S6). The results highlighted that there were unknown *Fusobacterium* members in the human gut, but future validations are still needed.

**Fig. 3:**
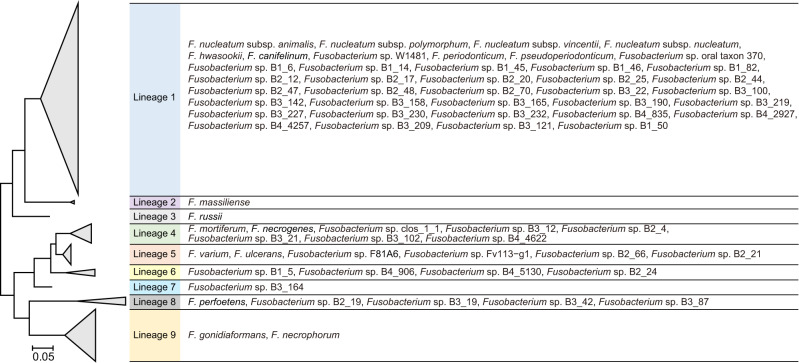
*Fusobacterium* had diverse species members and could be divided into nine phylogenetic lineages. Newly identified and previously known species (except *F. naviforme*) were included. The tree was based on the corresponding sequences in the genomes or obtained via FrpoB-seq. Branches of the same lineages are compressed with lineage names shaded in different colours, and the names of species belonging to those lineages are listed accordingly. See also Fig. S5 for the full tree.

Meanwhile, the phylogeny based on the *rpoB* region indicated that all *Fusobacterium* species could be categorised into nine lineages (designated L1–L9) (Figs. 3 and S5). This lineage classification was consistently observed in the ANIb matrix-based dendrogram, the phylogenetic trees of 16S rRNA genes, full-length *rpoB* and the selected *rpoB* regions of the studied genomes, as well as, the tree of the *rpoB* sequences obtained by FrpoB-seq (Figs. S3 and S7), implying that could be an important genetic feature of *Fusobacterium*.

### Compositional features of the *Fusobacterium* communities in colorectal cancer tissues

**Fig. 4:**
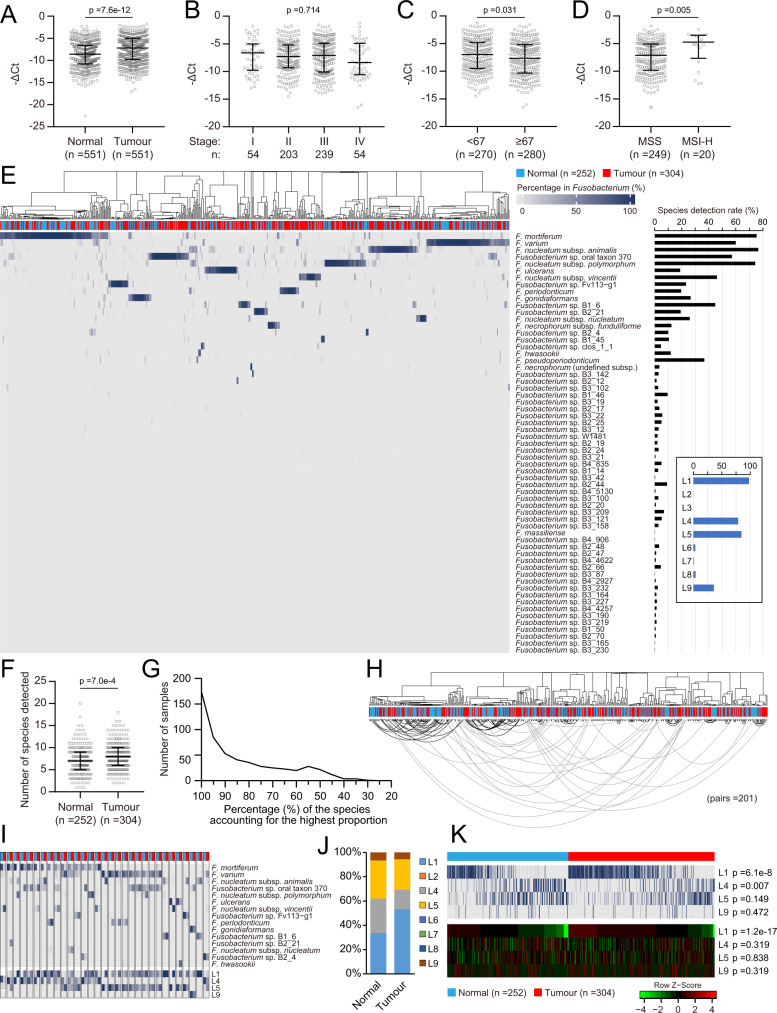
Compositional features of the *Fusobacterium* communities in colorectal cancer tissues. **A** Relative abundance of total *Fusobacterium* among bacteria in tumour and adjacent normal tissues. The *p* value of Mann–Whitney test is shown while that of Wilcoxon matched-pair signed-rank test is 8 × 1e–30. **B** Relative abundance of *Fusobacterium* in tumour tissues of different stages. Kruskal–Wallis test followed by Dunn’s multiple comparison test. The *p* value of Kruskal–Wallis test is shown. **C** Relative abundance of *Fusobacterium* in tumour tissues of different age groups separated by the median age. Mann-Whitney test. **D** Relative abundance of *Fusobacterium* in tumour tissues grouped by MSI status. Mann–Whitney test. **E** *Fusobacterium* species detected in tumour and normal tissues. Their percentages in the *Fusobacterium* community of each sample are presented as a heatmap. The black or blue histogram on the right shows the detection rate of each species or defined lineage in the samples, respectively. **F** Numbers of detected species in tumour and normal tissues. Mann–Whitney test. **G** Distribution of the percentage of the most abundant *Fusobacterium* species in each sample. **H** Dendrogram based on the *Fusobacterium* species compositions (Fig. S9) of 201 paired tumour and normal tissues. Paired samples are connected by lines. The grey lines indicate the paired samples located on separate major branches while black lines indicate otherwise. **I** *Fusobacterium* species compositions showing different patterns in paired tumour and normal tissues. The colour gradient scheme is the same as that in (**G**). Species with a <5% proportion are not shown. **J** Overall *Fusobacterium* lineage compositions in the tumour and normal tissues. L, lineage. **K** Percentages in *Fusobacterium* communities (upper heatmap) and estimated relative abundance among bacteria (lower heatmap) of the defined lineages compared between tumour and normal tissues. Lineages with low detection rates were not included; Mann-Whitney test followed by the Benjamini-Hochberg correction. Adjusted *p* values are shown. For **A**–**D** and **F**, individual data points are shown along with the medians and interquartile ranges. All statistical analyses are two-sided where applicable. Source data are provided as a source data file and in supplementary data 3.

To investigate the specific roles of various *Fusobacterium* members in CRC, the compositional features of *Fusobacterium* communities in the tissues of CRC patients were further analysed. Quantification with the designed *rpoB*-targeted primers showed that the relative *Fusobacterium* abundance among the total bacterial population was significantly higher in tumour tissue than in the normal mucosa (Fig. 4A). The abundance in tumours showed no difference across stages (Figs. 4B and S8). However, elderly patients (≥67 years old) had lower *Fusobacterium* abundance in tumours than younger patients (Fig. 4C). In addition, higher *Fusobacterium* loads were observed in microsatellite instability-high (MSI-H) tumours than in microsatellite stable (MSS) tumours (Fig. 4D). The tumoural *Fusobacterium* abundance showed no association with other analysed pathological features, including the *KRAS* mutation status and EGFR or p53 immunohistochemical (IHC) staining results (Fig. S8).

Next, we study the patterns of *Fusobacterium* communities with the FrpoB-seq data (Supplementary Data 3). We found that the *Fusobacterium* communities in both tumour (*n* = 304) and normal (*n* = 252) tissues displayed surprisingly diverse compositional patterns at the species level (Fig. 4E). In total, 60 species were identified in those samples, with varied detection rates. Notably, in addition to the canonical taxon *F. nucleatum*, *Fusobacterium mortiferum*, *Fusobacterium varium*, and *Fusobacterium* sp. oral taxon 370 were also prevalent. Among the *F. nucleatum* “subspecies”, *animalis* and *polymorphum* were highly prevalent, while *nucleatum* showed the lowest detection rate. At the lineage level, L1, L4, L5 and L9 were prevalent, and L2, L5, L6 and L7 were rare, while L3 was not detected. Between one and 20 species (median: seven) were detected in each sample, and the tumours contained more species than the normal mucosa (Fig. 4F). Strikingly, the *Fusobacterium* community in a sample was generally predominated by one of its species members; in over 96% of the samples, the most abundant species accounted for >50% of the total *Fusobacterium* community (Fig. 4G).

The *Fusobacterium* species compositional patterns in tumours did not show clear differences but rather were similar to those in the corresponding adjacent normal mucosa. Among the 201 pairs of samples for which FrpoB-seq was successfully applied, 170 (84.6%) had compositional patterns clustered within sister edges of the dendrogram or at least the same major clade (Figs. 4H and S9). Interestingly, in the remaining 31 pairs with distinct patterns, the predominant species exhibited an apparent shift from non-L1 lineages in the normal mucosa to the L1 lineage in tumours (Fig. 4I).

We next used the FrpoB-seq data for tumour and normal mucosa tissues to probe individual *Fusobacterium* members associated with CRC. Statistical analysis at the species level was not particularly productive due to the wide variation in species composition among samples, especially because the predominance of a particular species, even a frequently detected one, was confined to a relatively small proportion of the samples. We, therefore, conducted the analysis at the lineage level instead. Notably, the proportions of L1 and L4 in *Fusobacterium* communities showed differences between the tumour and normal mucosa samples (Fig. 4J, K). After incorporating qPCR data for total *Fusobacterium* quantification, we found that L1 was the only lineage that showed a difference in relative abundance among the total bacterial population, which was significantly increased in tumours (Fig. 4K).

**Fig. 5:**
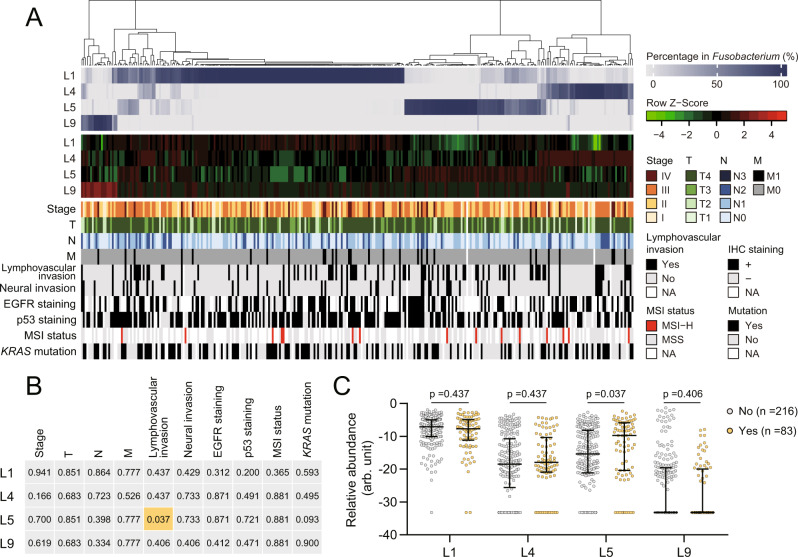
Lineage 5 was enriched in tumour samples associated with lymphovascular invasion. **A** A heatmap showing the percentages in *Fusobacterium* communities and estimated relative abundance among bacteria in tumours and corresponding pathological characteristics were included for analysis. **B** Associations between lineage abundance and pathological characteristics. For each pathological characteristic, patients were grouped by the corresponding categories listed in (**A**), and lineage abundance was compared. The *p* values are summarised with orange and grey shades denoting significant and insignificant results. See (**C**) and Fig. S10 for details of comparison and statistical analyses. **C** L5 abundance showed an association with lymphovascular invasion. Mann-Whitney test (two-sided) followed by the Benjamini-Hochberg correction. Adjusted *p* values are shown. Individual data points are shown along with the medians and interquartile ranges. arb. unit, arbitrary unit. See Supplementary Data 3 and  4 for detailed data.

We further assessed whether the *Fusobacterium* lineages in tumours were associated with particular pathological characteristics (Fig. 5A and Supplementary Data 4). The abundance of L1, L4 or L9 in tumours did not display an association with the examined pathological features (Figs. 5B and S10). In contrast, the abundance of L5 in tumours, though not different from that in the paired normal mucosa, exhibited a strong positive association with lymphovascular invasion (Fig. 5B, C). The above results uncovered that *Fusobacterium* had lineage-specific correlations in CRC.

### Compositional features of the *Fusobacterium* communities in faecal samples from colorectal cancer patients

**Fig. 6:**
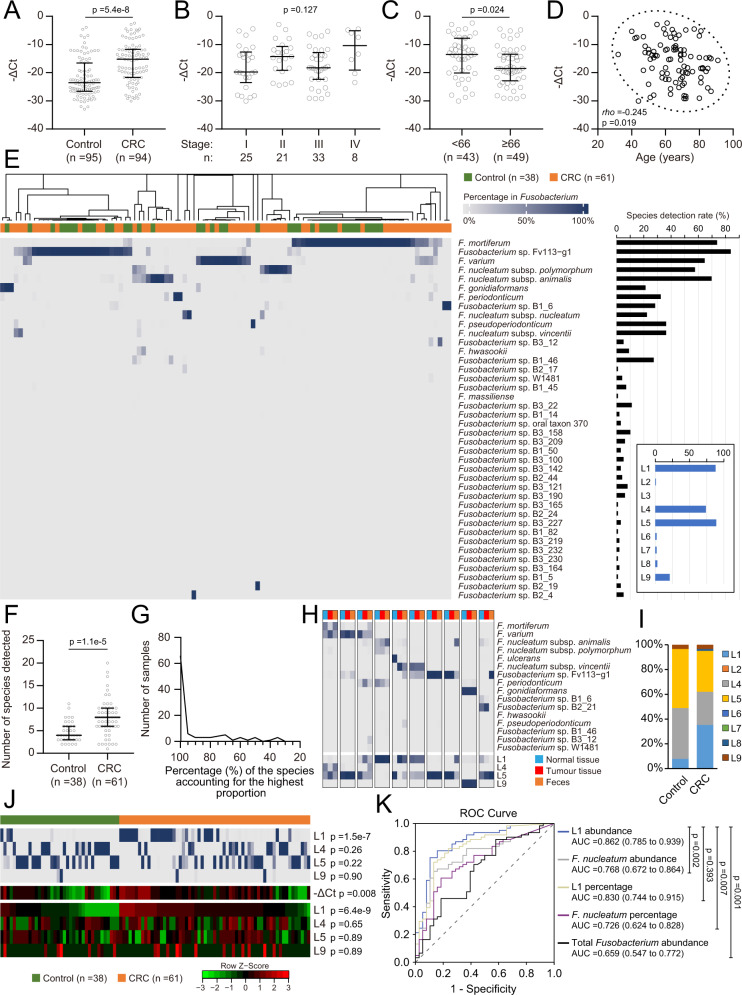
FrpoB-seq identified similar *Fusobacterium* patterns in faeces and that lineage 1 abundance was predictive of CRC. **A** Relative abundance of total *Fusobacterium* among bacteria in faecal samples from colorectal cancer (CRC) patients and healthy controls. Mann–Whitney test. **B** Relative abundance of *Fusobacterium* in faecal samples from CRC patients at different stages. Kruskal–Wallis test was followed by Dunn’s multiple comparison test. The *p* value of Kruskal–Wallis test is shown. **C** Relative abundance of *Fusobacterium* in faecal samples from CRC patients in different age groups separated by the median age. Mann–Whitney test. **D** Correlation analysis between the relative abundance of *Fusobacterium* in faecal samples and patient age. Spearman correlation test. **E** *Fusobacterium* species compositions were detected in the faecal samples from CRC patents and healthy controls. The black or blue histogram on the right indicates the detection rate of each species or defined lineage, respectively. **F** The number of detected species in faecal samples. Mann–Whitney test. **G** Distribution of the percentage of the most abundant species in each sample. **H** *Fusobacterium* species compositions in faecal samples and matching normal and tumour tissues. Species with a <5% proportion are not shown. L, lineage. **I** Overall *Fusobacterium* lineage compositions in the CRC and control faecal samples. **J** Percentages in the *Fusobacterium* community (upper heatmap) and estimated relative abundance among bacteria (lower heatmap) of the defined lineages compared between CRC and control. The scale in **E** is applicable to the percentage heatmap. Lineages with low detection rates were not included. The Mann–Whitney test followed by the Benjamini–Hochberg correction was used, and adjusted *p* values are shown. **K** Receiver operating characteristic (ROC) curve for predicting CRC. AUC, the area under the ROC curve. In brackets are the 95% confidence intervals. The data were obtained from the participants in (**E**). Pairwise comparisons of ROC curves were conducted with MedCalc (the DeLong method) followed by the Benjamini–Hochberg correction, and adjusted *p* values are shown. For (**A**)–(**C**) and (**F**), medians and interquartile ranges are shown. All statistical analyses are two-sided where applicable. Source Data are provided as a source data file and in Supplementary data 3.

Next, we studied the compositional features of *Fusobacterium* communities in faecal samples. Notably, the results were consistent with the findings in tissues. Higher *Fusobacterium* loads were found in the faeces of CRC patients than in that of controls (Fig. 6A). The *Fusobacterium* abundance among the total bacterial population in the faeces of CRC patients showed no association with the stage (Figs. 6B and S11) but displayed a weak but significant negative correlation with age (Fig. 6C, D). FrpoB-seq of 99 samples revealed 40 species with varied detection rates (Supplementary Data 3 and Supplementary Data 5), which were generally consistent with the distribution in tissue samples (Fig. 6E). More species were detected in the faeces of CRC patients than in that of the controls (Fig. 6F). The apparent feature of one-species predominance in the samples was maintained in the faeces (Fig. 6G). Compositional patterns at the species level between CRC and control samples could still not be differentiated. In addition, the compositions in faeces generally showed consistent patterns to those in the paired tissue samples, as observed in patients with available FrpoB-seq data for tissue and faecal samples (Figs. 6H and S12). In addition, consistent with the tissue-derived results, L1 was found at higher levels in terms of both percentage in the *Fusobacterium* community and relative abundance among the total bacterial population in CRC samples than in control samples (Fig. 6I, J). Importantly, L1 abundance exhibited predictive value for CRC (AUC = 0.862), performing better than the percentage or abundance of the canonical taxon *F. nucleatum* or the abundance of total *Fusobacterium* (Fig. 6K). These results further supported that L1 was associated the development of CRC.

## Discussion

This study clarified the taxonomy of *Fusobacterium*, devised a *Fusobacterium* detection method that enabled accurate profiling of the species composition of gut *Fusobacterium* in CRC patients and revealed that *Fusobacterium* members may play distinct but lineage-specific roles in CRC.

Genotyping approaches have greatly facilitated the clarification of *Fusobacterium* taxonomy^3,14,15,17^. However, concerns have recently been raised regarding the taxonomic rank of the subspecies of *F. nucleatum*. Due to the sufficient divergence revealed by genomic analyses, several studies have proposed that the subspecies should be considered as separate species^14,15^. Our comprehensive taxonomic analyses conclusively indicate that the “subspecies” are indeed separate species. Although most studies, including our previous study^20^, regard *F. nucleatum* as one species, as all the subspecies have been isolated from and detected in CRC tumours and collectively display an association with CRC^3^, we show that the tumorigenic features do not justify grouping the subspecies as one species, since these features are actually exhibited by a wider spectrum of species belonging to the same lineage.

We further found that *rpoB-*based analysis could distinguish *Fusobacterium* species at fine resolution and applied this finding by developing the FrpoB-seq method, which allowed us to easily and stringently profile *Fusobacterium* species in microbiota-containing samples. Using FrpoB-seq, we were able to achieve high-depth profiling of this genus, which allowed us to uncover undescribed low abundance species. There were a striking number of previously undescribed species residing in the human gut. The diversity of *Fusobacterium* species in the gut microbiota is much higher than previously known. The compositions of gut *Fusobacterium* communities are seemingly stable within individuals but differed widely among individuals. This phenomenon implies that there might be multiple species involved in CRC, rather than one particular species. Indeed, we found that the L1 lineage which includes *F. nucleatum*, *F. periodonticum* and *F. pseudoperiodonticum* was overabundant in CRC, while L5 which includes *F. varium* and *Fusobacterium ulcerans* was associated with lymphovascular invasion. These results indicate that *Fusobacterium* members may have distinct but lineage-specific pathogenic behaviours in CRC, which might be attribute to the different distributions of putative invasion-associated virulence genes in theses lineages^15^. Thus, the L1 and L5 can be considered as different biomarkers or targets in CRC.

This research had some limitations. The suitability of FrpoB-seq for samples containing undetectable levels of or no *Fusobacterium* may be limited. Additionally, FrpoB-seq does not cover *F. naviforme*. However, the taxonomic position of *F. naviforme* remains in doubt^21^, and it may not be a member of the *Fusobacterium* genus and likely to be rare in the human gut^12^. Also, this was a single-centre study focused on CRC. To expand the understanding of the distribution of *Fusobacterium* members in humans and their associations with CRC as well as other diseases, large-scale studies on different populations with different disease statuses are required.

In conclusion, with the FrpoB-seq approach, we provide a high-resolution view of the gut *Fusobacterium* communities in CRC patients, uncover a considerable number of undescribed *Fusobacterium* species and reveal that *Fusobacterium* members may play distinct lineage-specific roles in CRC. This study can help to clarify the association between gut *Fusobacterium* members and CRC and precisely probe the oncogenic culprit. We envision that FrpoB-seq will serve as a useful tool aiding future studies.

## Methods

### Ethics

This study was approved by the Ethics Committee of Shanghai Tenth People’s Hospital (No. SHSY-IEC-4.1/20-85/01). Written consent was obtained from each participant.

### Bacterial strains

The bacterial strains used in this study are listed in Table S1. Nineteen *Fusobacterium* strains were isolated from tumour tissues as previously described^10^. Tumour tissues were placed in tryptic soy broth (#11104, Beijing SanYao) with 0.05% cysteine-hydrochloride (C7352, Sigma-Aldrich), disaggregated and plated on isolation media. Multiple media were used in parallel for isolation, including Crystal Violet Erythromycin Agar (CVE)^22^; *Fusobacterium* egg yolk agar (FEA)^23^; *Fusobacterium* Selective Agar (T1141, Rui Chu Biotech) supplemented with 5% defibrinated sheep blood (Shanghai HuaKang); josamycin/vancomycin/norfloxacin (JVN) agar^24^; brain-heart infusion agar (HB8478, Hope Biotech) supplemented with crystal violet, neomycin sulphate and vancomycin (8.75, 37.5 and 6.25 μg/ml, respectively, Sangon Biotech); and Columbia blood agar (Comagal Microbial). The 16S rRNA genes were sequenced with the Sanger method for species identification. All strains were cultivated on Columbia blood agar at 37 °C in a jar containing an AnaeroPack System (C-1, Mitsubishi Gas Chemical).

### Clinical samples

For *Fusobacterium* isolation, freshly resected tumour tissues were collected from ten CRC patients. For species-level *Fusobacterium* characterisation, fresh-frozen tumour and adjacent normal tissue samples from 551 primary CRC patients receiving surgical resection and faecal samples from 94 primary CRC patients receiving surgical resection (>18 years old, either sex) were retrieved from the Biobank of Shanghai Tenth People’s Hospital. Among the patients, there were 29 who had both tissue and faecal samples collected and characterised. Faecal samples were also collected from 95 healthy volunteers. Patients who had received antibiotics or probiotics within one month prior to sample collection were excluded. Clinical and pathological information of patients was obtained from their medical records. Demographic information was collected from healthy volunteers.

### Complete sequencing of bacterial genomes

Seven of the *Fusobacterium* isolates including *Fusobacterium hwasookii* strain THCT14E2, *F. nucleatum* subsp*. polymorphum* strains THCT7E2 and THCT15E1, *F. nucleatum* subsp. *vincentii* strain THCT14B3 and *F. nucleatum* subsp. *animalis* strains THCT5A4, THCT6B3 and THCT7A2, were sequenced with the PacBio RSII platform (350×–500× coverage) combining the Illumina HiSeq 4000 platform (~450× coverage) at Beijing Genomics Institute (BGI), Shenzhen. PBdagcon (https://github.com/PacificBiosciences/pbdagcon) was used for subread correction. GATK v1.6 (https://github.com/broadinstitute/gatk) and the SOAP tool package^25^ were used for single-base correction. Genomes were assembled with Celera Assembler v8.3 (http://wgs-assembler.sourceforge.net). Coding genes were predicted with Glimmer v3.02^26^, while non-coding genes were annotated with RNAmmer v1.2^27^ and tRNAscan-SE v1.31 ^28^.

### Public bacterial genomic data

A total of 192 *Fusobacterium* genomes were retrieved from the NCBI database (ftp://ftp.ncbi.nih.gov/genomes/). Duplicated genomes or those not meeting the RefSeq criteria were excluded. Finally, 150 unique genomes, including 40 complete and 110 draft genomes, were included for analysis (Supplementary Data 1).

### Average nucleotide identity analysis

The sequenced and downloaded genomes (*n* = 157) were subjected to pairwise whole-genome average nucleotide identity calculated with BLAST (ANIb) analysis with JSpecies v1.2.1^18^. Notably, each pair of genomes was examined twice, using one genome as the query and the other as the subject and vice versa, which might have generated two slightly different values due to the nature of the algorithm.

### Multiple sequence alignment and phylogenetic analysis

Multiple sequence alignments were performed with MUSCLE^29^, and phylogenetic trees were subsequently constructed with MEGA5^30^ using the maximum likelihood algorithm and 1000 bootstrap replicates. Whole-genome based phylogenetic analysis was conducted with kSNP3 ^31^.

### Quantitative polymerase chain reaction

Total DNA from the faeces and tumour tissues was prepared with the cetyltrimethylammonium bromide (CTAB)-based method. The abundance of *Fusobacterium* was quantified by quantitative polymerase chain reaction (qPCR) with the primers fuso-rsub-F2 (5′-GCCTCATTTTYTDGARTTYCAATT-3′) and fuso-rsub-R2 (5′-ACDACTCTTTCHGCHCCATTKAT-3′), which were designed in this study to target the *Fusobacterium rpoB* gene. The reference was the bacterial 16S rRNA gene, which was quantified with a combination of five primers used together (5′-CNACGCGAAGAACCTTANC-3′, 5′-ATACGCGARGAACCTTACC-3′, 5′-CTAACCGANGAACCTYACC-3′, 5′-CAACGCGMARAACCTTACC-3′, and 5′-CGACRRCCATGCANCACCT-3′)^32^. Primers were synthesized by Generay Biotech (Shanghai). qPCR was conducted with a TB Green Premix Ex Taq II kit (RR820, Takara) on a 7500 Real-Time PCR system (Applied Biosystems) according to the manufacturer’s instructions. In each reaction, 90 ng of DNA was used. The thermal cycling conditions were 50 °C for 2 min, 95 °C for 30 s, and 35 cycles of 95 °C for 5 s, 50 °C for 30 s and 70 °C for 30 s. If *Fusobacterium* was not detected, 45 cycles were then applied. A ΔCt value was calculated for each sample. Human blood DNA from healthy volunteers and total DNA of *F*. *nucleatum* subsp. *nucleatum* ATCC 25586 were used as negative and positive controls, respectively. A faecal sample tested to be negative for *Fusobacterium* was also used as a negative control. To assess the amplification performance of fuso-rsub-F2/R2, standard PCR was performed with a Premix Taq™ kit (RR903, TAKARA) using the conditions same as above. The primers Fn-F (5′-CAACCATTACTTTAACTCTACCATGTTCA-3′ and Fn-R (5′-GTTGACTTTACAGAAGGAGATTATGTAAAAATC-3′) widely used for *F. nucleatum* detection were also used for comparison^5,33^. For qPCR, a repeat was made for each sample, while for standard PCR, experiment was performed in triplicate.

### Fusobacterium rpoB amplicon sequencing

FrpoB-seq was conducted with the primers fuso-rsub-F2 and fuso-rsub-R2 at BGI, Shenzhen, following a standard amplicon sequencing procedure. For amplification, 30 ng of DNA extracted from a clinical sample was used in each reaction. The PCR conditions were 95 °C for 3 min; 35 cycles of 95 °C for 30 s, 50 °C for 45 s and 72 °C for 45 s; and 72 °C for 10 min. High-throughput sequencing was performed on the Illumina HiSeq 2500 platform. Paired-end reads with an overlap of ≥15 bp and a mismatch rate of <0.1 were assembled with FLASH v1.2.11^34^. Operational taxonomic units (OTUs) were generated with 100% identity via USEARCH (https://drive5.com/usearch/). Multiple sequence alignment of the OTUs based on the *rpoB* amplicon sequences and the corresponding *rpoB* regions from the 157 genomes was conducted, followed by phylogenetic tree construction; this phylogenetic tree was used to annotate the OTUs at the species level. The percentages of the derived species in the *Fusobacterium* communities were calculated based on the FrpoB-seq data. To further estimated the relative abundance of each species in the microbiota, the FrpoB-seq-derived percentages were normalised to the relative abundance of total *Fusobacterium* determined by qPCR. Serially diluted (10×) mock samples containing nine *Fusobacterium* species and subspecies (Table S1) with available strains were firstly used to assess the minimum overall *Fusobacterium* load allowing successful construction of a standard sequencing library. Then, mock samples containing serially diluted *F. nucleatum* subsp*. nucleatum* or *F. varium* supplemented by *F. mortiferum* were used to assess the sensitivity of FrpoB-seq. A mock sample containing defined percentages of the nine species and subspecies (Fig. S4B) was also used to validate the amplification performance.

### Metagenomic sequencing and public metagenomic data

Metagenomic sequencing of faecal samples were conducted at BGI, Shenzhen, following a standard procedure. For library construction, 1 μg DNA was used. High-throughput sequencing was performed on the BGI DNBSEQ platform at an average ~10 Gbp depth. Contigs were assembled with MEGAHIT^35^, and then used to search for undescribed *Fusobacterium* species. A detailed pipeline is shown in Fig. S6. Publicly available metagenomic data were retrieved from the NCBI SRA database and the accessions were PRJNA557323 for the Southern Chinses population cohort and PRJNA678426 for the ultra-deep sequencing (Korean population) cohort^12,19^.

### Heatmaps

Heatmaps were generated with the gplots (https://github.com/talgalili/gplots) or ComplexHeatmap^36^ package in R, as applicable. The accompanying dendrograms were drawn with default parameters.

### Statistics

Data were analysed with GraphPad Prism (version 5), SPSS (version 19) or R (version 4.0.2). The Mann–Whitney test was used for comparisons between two groups. The Wilcoxon signed-rank test was used to analyse paired data. For comparisons among multiple groups, the Kruskal–Wallis test followed by Dunn’s multiple comparison test was used. To identify taxa that were differentially abundant between two groups from the FrpoB-seq data, the Mann–Whitney test followed by the Benjamini–Hochberg correction was used. The Spearman rank correlation test was used for correlation analysis. Pairwise comparisons of ROC curves were conducted with MedCalc (the DeLong method). A two-sided *p* < 0.05 was considered statistically significant.

### Reporting summary

Further information on research design is available in the Nature Research Reporting Summary linked to this article.

## Supplementary information

Supplementary Information

Description of Additional Supplementary Files

Supplementary Data 1

Supplementary Data 2

Supplementary Data 3

Supplementary Data 4

Supplementary Data 5

Reporting Summary

## Source data

Source Data

## Data Availability

The data generated or analysed during this study are included within the paper, its Supplementary information/data files, and public repositories. The bacterial genome data generated in this study have been deposited in the NCBI GenBank database under the accession CP071099 for strain THCT5A4, CP071098 for THCT6B3, CP071097 for THCT7A2, CP071096 for THCT7E2, CP071093 for THCT14A3, CP071092 for THCT14E2 and CP071094–CP071095 for THCT15E1. Other raw sequencing data generated in this study have been deposited in the NCBI SRA database under the accession number PRJNA715828. Detailed information of 192 publicly available *Fusobacterium* genomes retrieved from the NCBI nucleotide database is listed in Supplementary data 1 with accession numbers included. The publicly available metagenomic datasets used were retrieved from the NCBI SRA database under the accessions PRJNA557323 and PRJNA678426. Source data are provided with this paper.
