# Author Correction: Profiling *Fusobacterium* infection at high taxonomic resolution reveals lineage-specific correlations in colorectal cancer

**DOI:** 10.1038/s41467-024-53491-z

**Published:** 2024-10-23

**Authors:** Dexi Bi, Yin Zhu, Yaohui Gao, Hao Li, Xingchen Zhu, Rong Wei, Ruting Xie, Chunmiao Cai, Qing Wei, Huanlong Qin

**Affiliations:** 1 Department of Pathology, Shanghai Tenth People’s Hospital, Tongji University School of Medicine, Shanghai, 200072 China; 2 Department of Gastrointestinal Surgery, Shanghai Tenth People’s Hospital, Tongji University School of Medicine, Shanghai, 200072 China

Correction to: *Nature Communications* 10.1038/s41467-022-30957-6, published online 9 June 2022

The original version of this Article contained an error in the primer sequences for fuso-rsub-F2 and fuso-rsub-R2 provided in the second sentence of the ‘Quantitative polymerase chain reaction’ section of the ‘Methods’, which incorrectly added some ‘Hs’. The correct version states ‘fuso-rsub-F2 (5’-GCCTCATTTTYTDGARTTYCAATT-3’)’ instead of ‘fuso-rsub-F2(5′-GCCTCATTTTHYTDGARTTYCAATT-3′)’ and ‘fuso-rsub-R2 (5’-ACDACTCTTTCHGCHCCATTKAT-3’)’ rather than ‘fuso-rsub-R2 (5′-ACDACTCTTTCHGCCCATTKHHAT-3′)’.

This has been corrected in both the PDF and HTML versions of the Article.

