# Supplementary material for: Profiling Fusobacterium infection at high taxonomic resolution reveals lineage-specific correlations in colorectal cancer
Source: Nat Commun. 2022 Jun 9;13:3336. doi: 10.1038/s41467-022-30957-6 (PMC9184491; doi:10.1038/s41467-022-30957-6)
Supplement: Supplementary file 1 — Supplementary Information [file 41467_2022_30957_MOESM1_ESM.pdf]

# Supplementary Information for

# Profiling *Fusobacterium* infection at high taxonomic resolution reveals lineage-specific correlations in colorectal cancer

Dexi Bi[1#]*, Yin Zhu[2#], Yaohui Gao[1#], Hao Li[2], Xingchen Zhu[1], Rong Wei[1], Ruting Xie[1], Chunmiao Cai[1], Qing Wei[1]*, Huanlong Qin[2]*

[1]Department of Pathology, Shanghai Tenth People's Hospital, Tongji University School of Medicine, Shanghai 200072, China

[2]Department of Gastrointestinal Surgery, Shanghai Tenth People's Hospital, Tongji University School of Medicine, Shanghai 200072, China

*Corresponding authors:

Dexi Bi. E-mail: bidexi@tongji.edu.cn.

Qing Wei. E-mail: weiqing1971@126.com.

Huanlong Qin. E-mail: qinhuanlong@tongji.edu.cn.

[#]These authors contributed equally to this study.

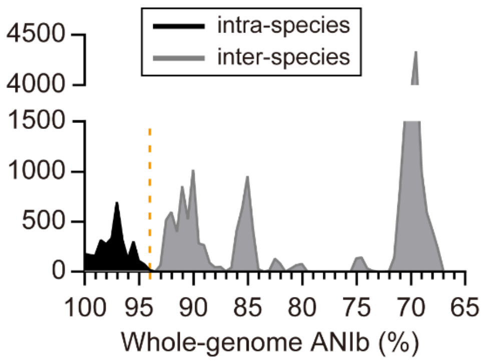


**Figure S1. Whole-genome ANIb analysis effectively defined species.** Distribution of pairwise intra-species (*n* =3,068) and inter-species (*n* =20,802) ANIbs of 157 *Fusobacterium* genomes is shown. Orange highlights species boundaries (94% ANIb). The analysis considered the four *F. nucleatum* subspecies as separate species. ANIb, average nucleotide identity calculated with BLAST.

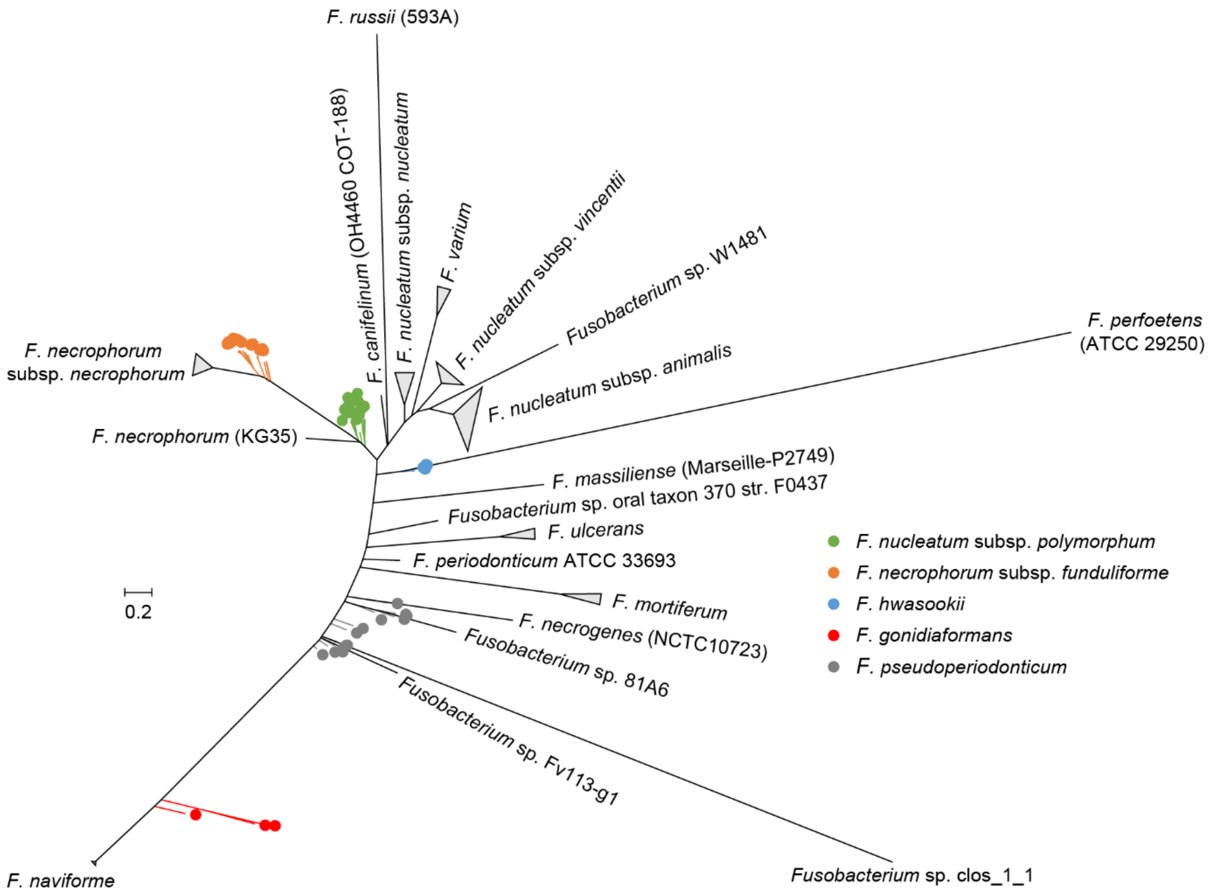


**Figure S2. Whole-genome phylogeny produced a *Fusobacterium* taxonomy similar to that produced by ANIb.** The 157 *Fusobacterium* genomes were used. The tree was generated by using kSNP3 with the maximum likelihood algorithm. Strain names are given in parentheses for the species with only one sequenced genome available. Branches of the same species/subspecies are compressed as applicable or denoted with dots of the same colour. The colour scheme is shown in the figure.

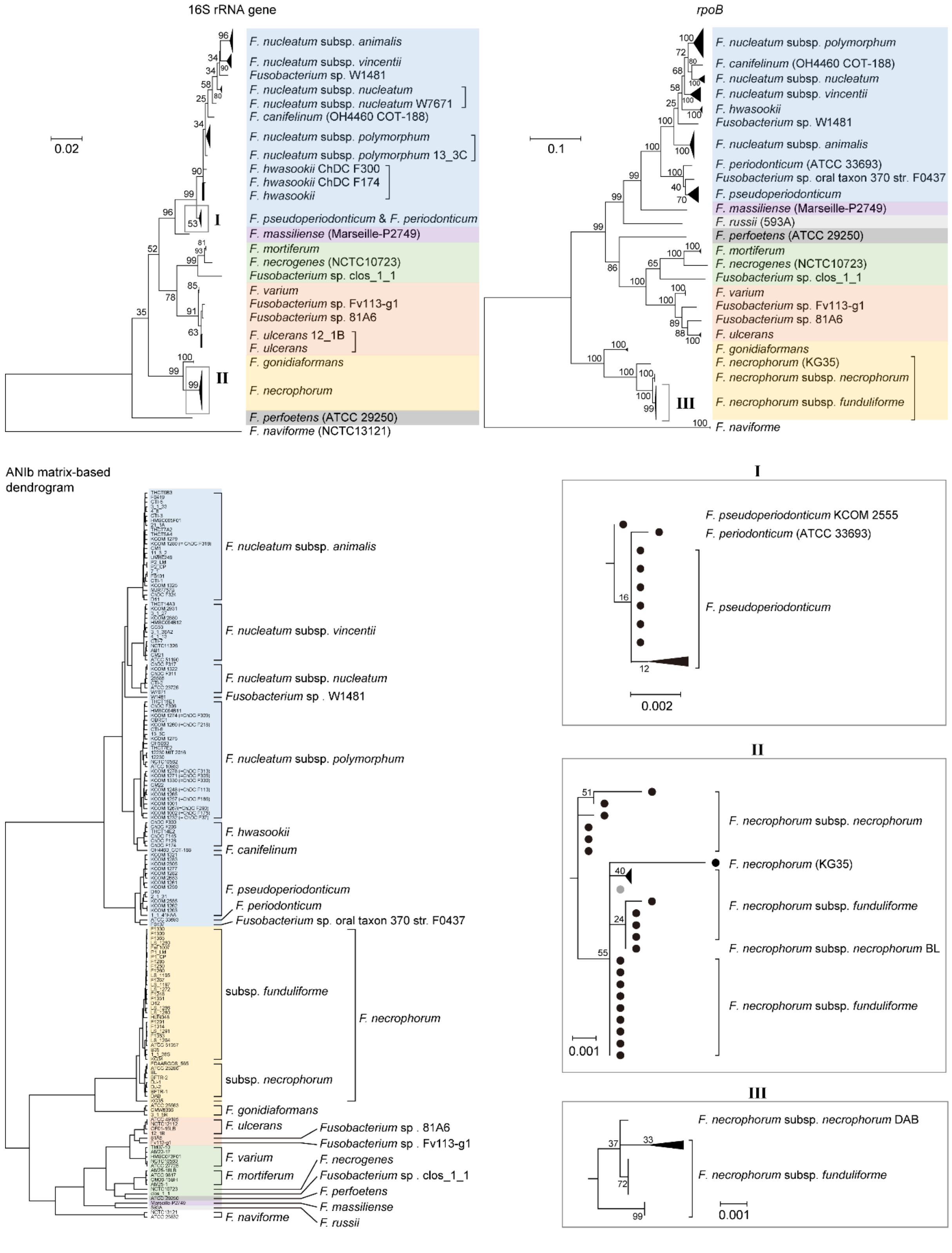


**Figure S3. Phylogenetic trees of the *Fusobacterium* 16S rRNA gene and *rpoB* were largely similar and the *rpoB*-based tree produced better delineation.** Complete gene sequences available in the 157 sequenced genomes were used for analysis (144 16S rRNA gene and 157

*rpoB* sequences). Strain names are given in parentheses for the species with only one sequence available. The I-III grey boxes correspond to the I-III labels in the trees. Strain names are also provided for those that could not be compressed together. Branches of the same species/subspecies or otherwise illustrated in the boxes are compressed as applicable. Stains on indistinctive edges are denoted by dots. The ANIb matrix-based dendrogram from Fig. 1 was also included for comparison. Species that could consistently form a lineage across the 16S rRNA gene tree, *rpoB* tree and ANIb-based dendrogram are shaded with the same colour. The colour scheme is identical to that used in Figs. 3, S5 and S7. ANIb, average nucleotide identity calculated with BLAST.

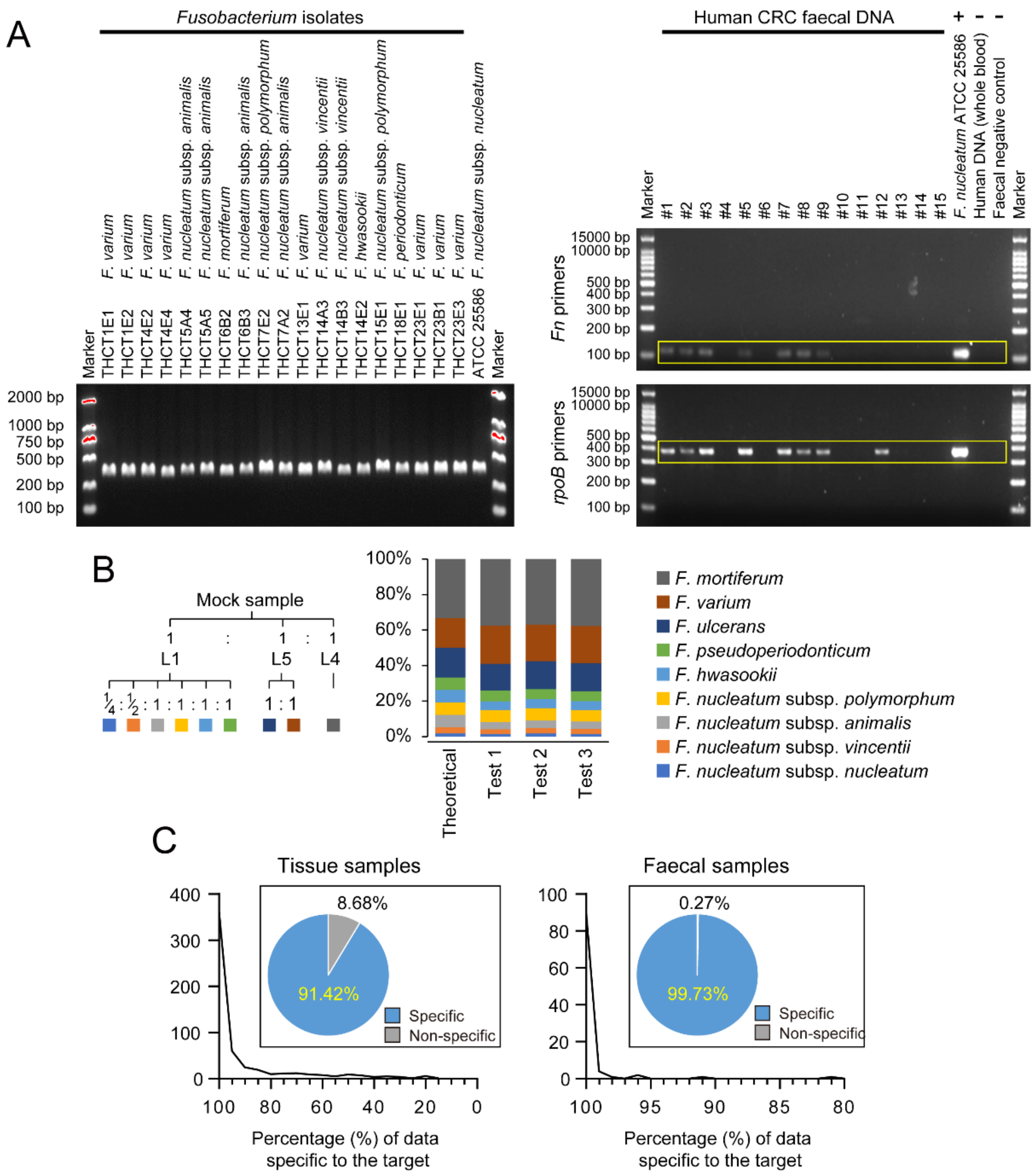


**Figure S4. Assessment of amplification performance.** (A) The designed primers amplified a specific band in *Fusobacterium* isolates and faecal samples from colorectal cancer (CRC) patients. For faecal samples, amplification with the *F. nucleatum* detection primers (*Fn* primers) was performed in parallel for reference. The *Fn* primers were the universal primers used for *F. nucleatum* detection (Fn-F: 5'-CAACCATTACTTTAACTCTACCATGTTCA-3' and Fn-R: 5'-GTTGACTTTACAGAAGGAGATTATGTAAAAATC-3') (Castellarin, *et al*, *Genome Res* 2012; Mima, *et al*, *JAMA Oncol* 2015), while the *rpoB* primers were the universal primers designed for the selected *rpoB* region. *F. nucleatum* subsp. *nucleatum* ATCC 25586 DNA was

used as a positive control ("+"). Human whole-blood DNA negative control and a faecal negative control were also used ("-"). Experiments were conducted in triplicate and representative gel images are shown. (B) Design of a mock sample and its composition detected by FrpoB-seq (in triplicate). (C) The FrpoB-seq data indicated high amplification specificity in the test samples. Distribution of the percentages of sequencing data specific to the *Fusobacterium rpoB* target in tissue and faecal samples are shown, with overall percentages given in the boxes. Colour schemes are denoted in the figure.

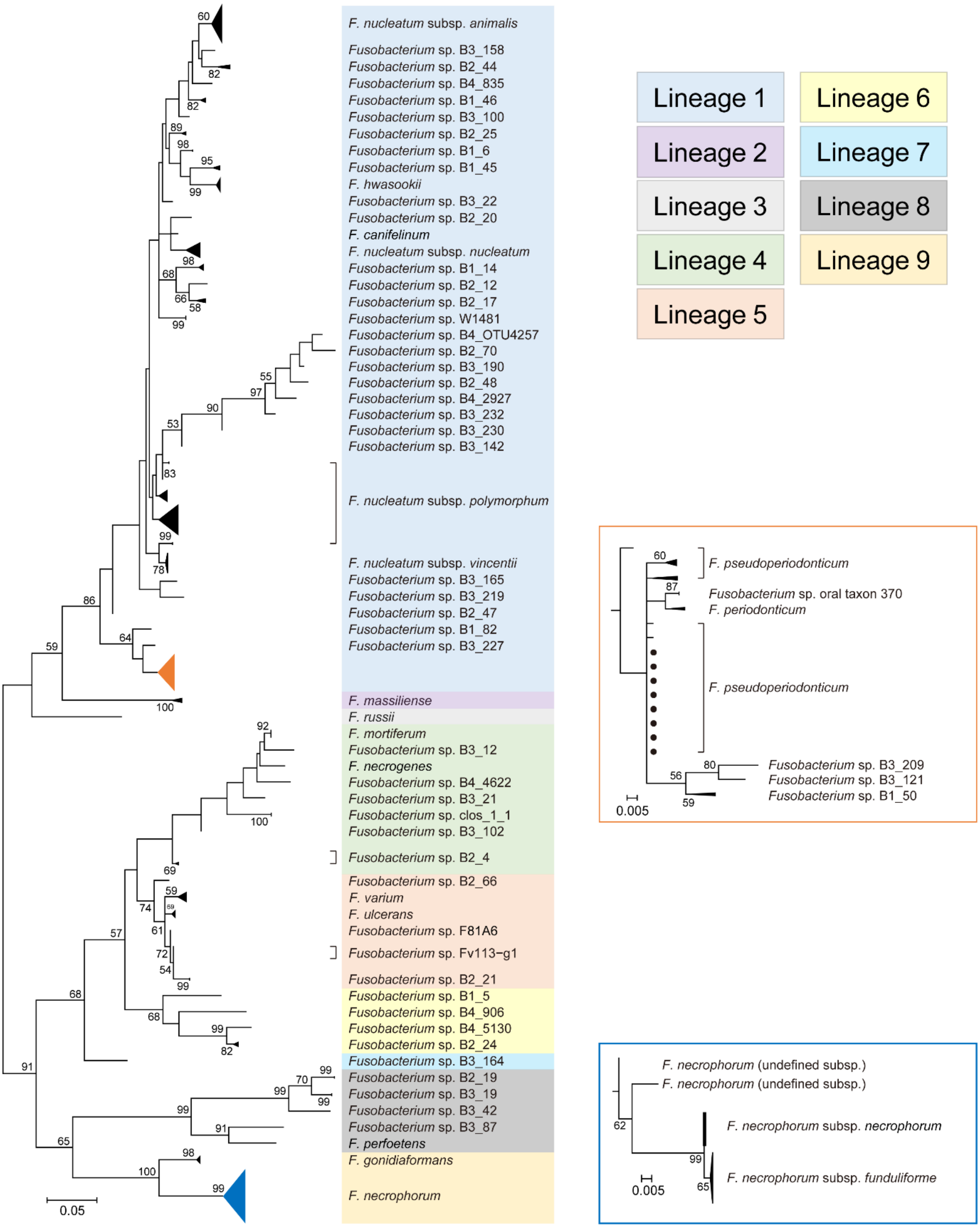


**Figure S5. The *rpoB*-based approach identified a striking number of new species and the phylogenetic tree of all *Fusobacterium* species based on the selected *rpoB* region delineated them into 9 lineages.** Both newly identified and previously known species were included in this analysis. The tree was based on the corresponding sequences in the genomes

or those obtained via FrpoB-seq. Branches of the same species/subspecies or those otherwise illustrated in the boxes are compressed as applicable. The boxes correspond to the triangles in the tree by same colours (orange and blue). Stains on indistinctive edges are denoted by dots. Lineages are denoted by different colours with the legend shown in the figure.

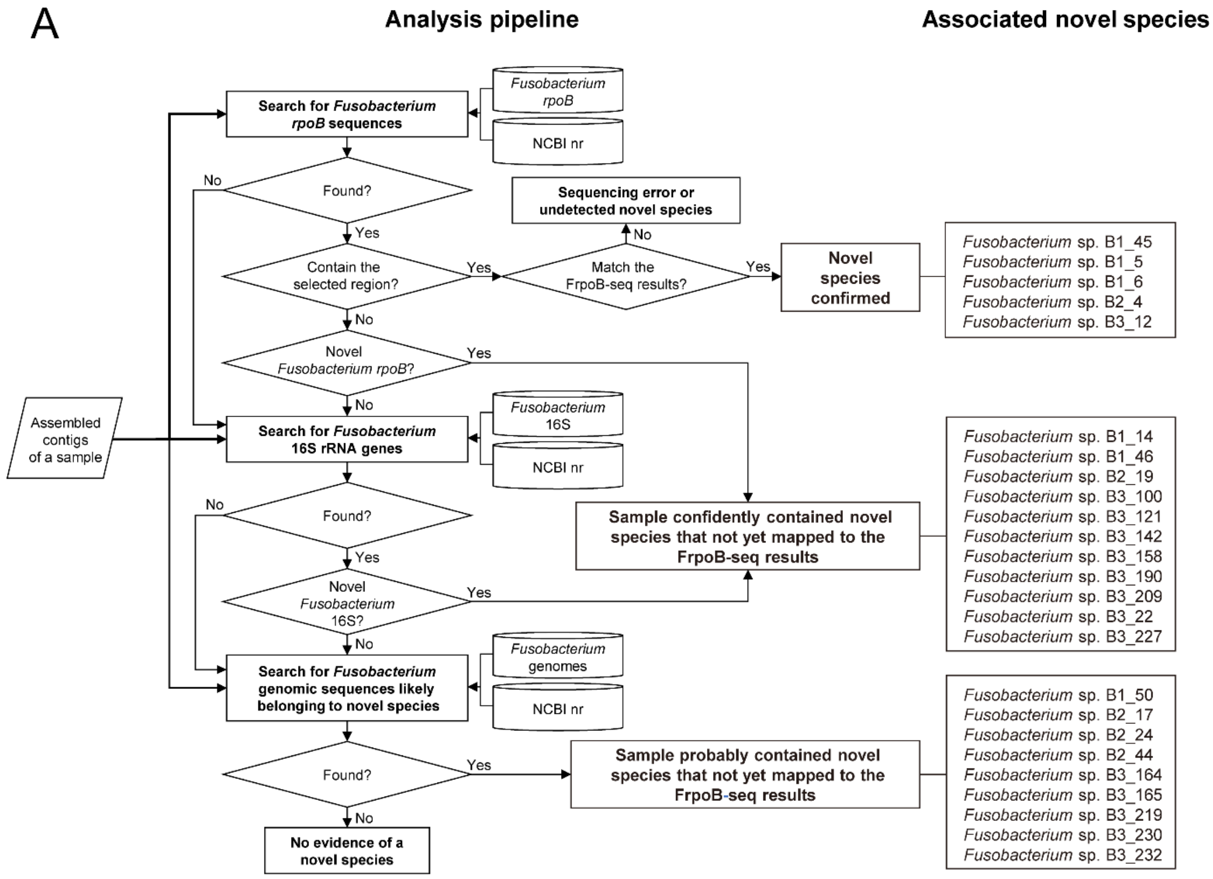


B

**Southern Chinese population cohort, n = 556 (Yeoh, *et al*, 2020)**
**(average ~7.5 Gbp)**

| **Accession(s)** | **Detected novel species** |
|---|---|
| SRR10680692 | *Fusobacterium* sp. B1_6 |
| SRR10680388, SRR10680416, SRR10680433, SRR10680435, SRR10680467, SRR10680471, SRR10680498, SRR10680545, SRR10680609, SRR10680651, SRR10680707, SRR10680730, SRR10680744, SRR10680761, SRR10680770, SRR10680788, SRR10901570 | *Fusobacterium* sp. B2_4 |
| SRR10680413 | *Fusobacterium* sp. B2_19 |
| SRR10680370, SRR10680429 | *Fusobacterium* sp. B2_24 |
| SRR10680577, SRR10680758, SRR10680776, SRR10680790, SRR10680868 | *Fusobacterium* sp. B3_19 |
| SRR10680530, SRR10680741 | *Fusobacterium* sp. B3_21 |
| SRR10680858 | *Fusobacterium* sp. B3_42 |
| SRR10680663 | *Fusobacterium* sp. B3_102 |
| SRR10680480 | *Fusobacterium* sp. B2_4, *Fusobacterium* sp. B2_19 |
| SRR10680611 | *Fusobacterium* sp. B2_19, *Fusobacterium* sp. B3_19 |
| SRR10680861 | *Fusobacterium* sp. B2_4, *Fusobacterium* sp. B3_19 |
| SRR10680767 | *Fusobacterium* sp. B3_19, *Fusobacterium* sp. B3_102 |
| SRR10680332, SRR10680691, SRR10680746 | *Fusobacterium* sp. B2_4 / *Fusobacterium* sp. B3_102 |
| SRR13061017 | *Fusobacterium* sp. B1_6 / *Fusobacterium* sp. B2_48 |
| SRR10680361 | putative novel species (~94% identity to *Fusobacterium* sp. B2_19) |
| SRR10680330 | putative novel species (~96% identity to *Fusobacterium* |
| SRR10680491 | putative novel species (~96% identity to *Fusobacterium* sp. B3_42) |

**Ultra-deep whole-metagenomic shotgun sequencing (Korea) cohort, n = 106 (Kim, *et al*, 2021)**
**(average >30 Gbp)**

| **Accession** | **Detected novel species** |
|---|---|
| SRR13061017 | *Fusobacterium* sp. B2_48 / *Fusobacterium* sp. B1_6 |

**Figure S6. Identification of putative novel *Fusobacterium* species with metagenomic sequencing data.** (A) Identification in a subset (n =35, see Dataset S3) of the collected faecal

samples covering 25 putative novel species. There were 26 putative novel species identified in faecal samples, but one (*Fusobacterium* sp. B1_82) could not be assessed due to that the sample containing it had insufficient DNA for sequencing. A three-step analysis pipeline was used. The assembled contigs of a sample were aligned with the full-length *Fusobacterium rpoB* and compared against the NCBI nucleotide (nr) database to search for *Fusobacterium*-specific *rpoB* sequences, which were then aligned with all available *rpoB* sequences (from the genomes and the FrpoB-seq data) to check if they could be mapped to those of the novel species. If the selected region used for FrpoB-seq was not covered in the metagenomic data, the available *rpoB* fragments were used to assess if they belonged to novel species at an identity cut-off of <96%, a species boundary found in Fig. 2A. *Fusobacterium*-specific 16S rRNA gene sequences were also search similarly and used to assess if they belonged to novel species at a cut-off of <98.5%, a stringent species boundary found in Fig. 2A. Finally, *Fusobacterium*-specific genomic sequences besides of *rpoB* and 16S rRNA gene, which had no non-*Fusobacterium* hit at a >20% coverage in the NCBI nr database, were search similarly and used to assess if they belonged to novel species. The criteria were that their identities to the hits of known species were <89% (a minus five of the ANIb boundary) with >50% coverages and also smaller than the intra-species identities of their alleles (if available). The identification results are given on the right side. Notably, scenario of sequencing error or existence of other novel species undetected by FrpoB-seq as denoted in the pipeline was not found. (B) Identification in public metagenomic datasets. *Fusobacterium*-specific *rpoB* sequences were selected and aligned against known sequences. The detected novel species that mapped to those identified by FrpoB-seq are given along with the corresponding sample accession numbers.

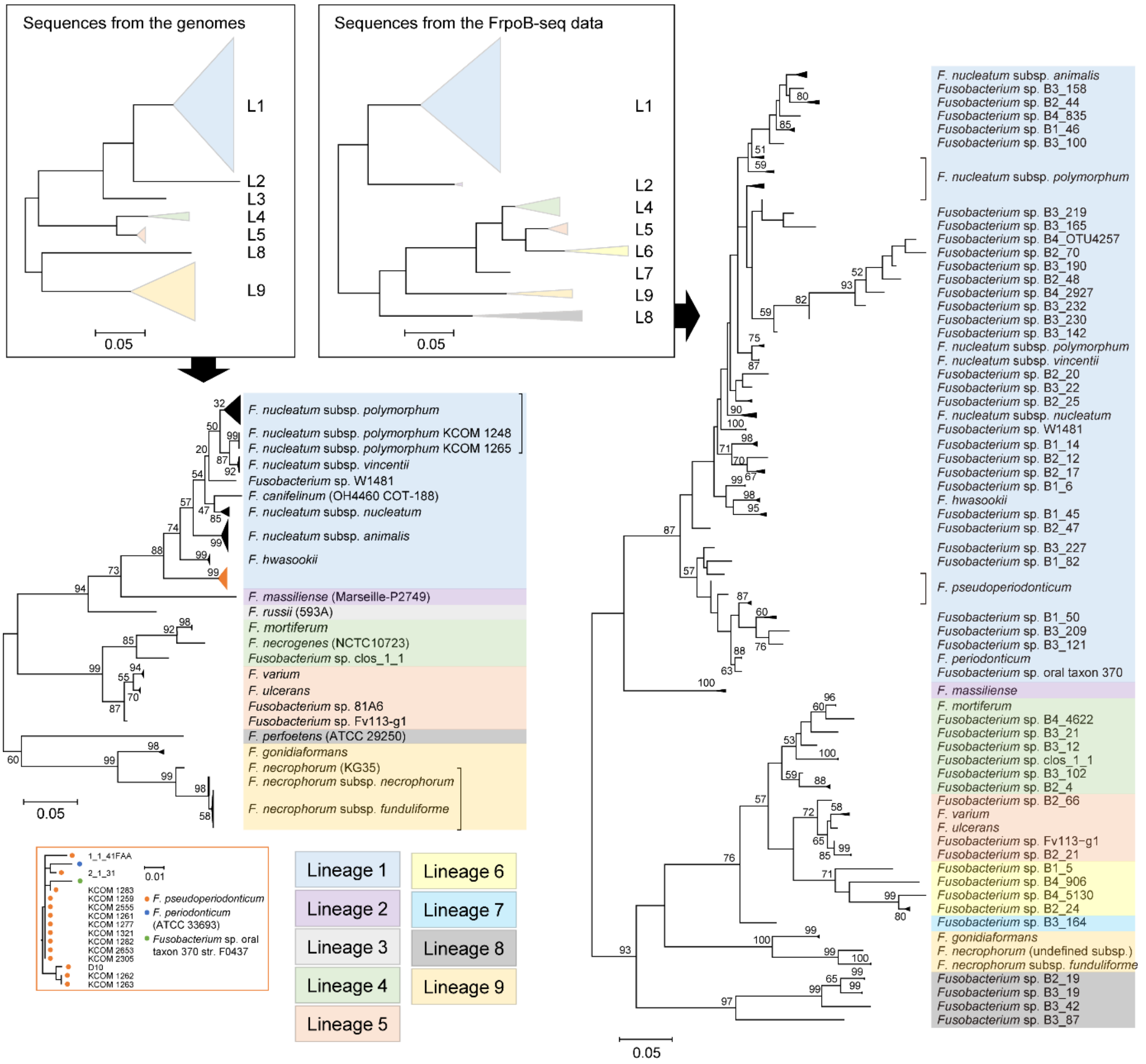


**Figure S7. The lineage classification results were concordant between the phylogenetic trees based on the sequences of the selected *rpoB* region from the genomes and the FrpoB-seq data.** Note that the tree of the former is identical to that shown in Fig. 2C. In that tree, branches of the same species/subspecies or those otherwise illustrated in the orange box (corresponding to the oragnge triangle). There was no available genome of L6 and L7 species, and in the FrpoB-seq data, no L3 species was detected. Lineages are denoted by different colours with the legend shown in the figure.

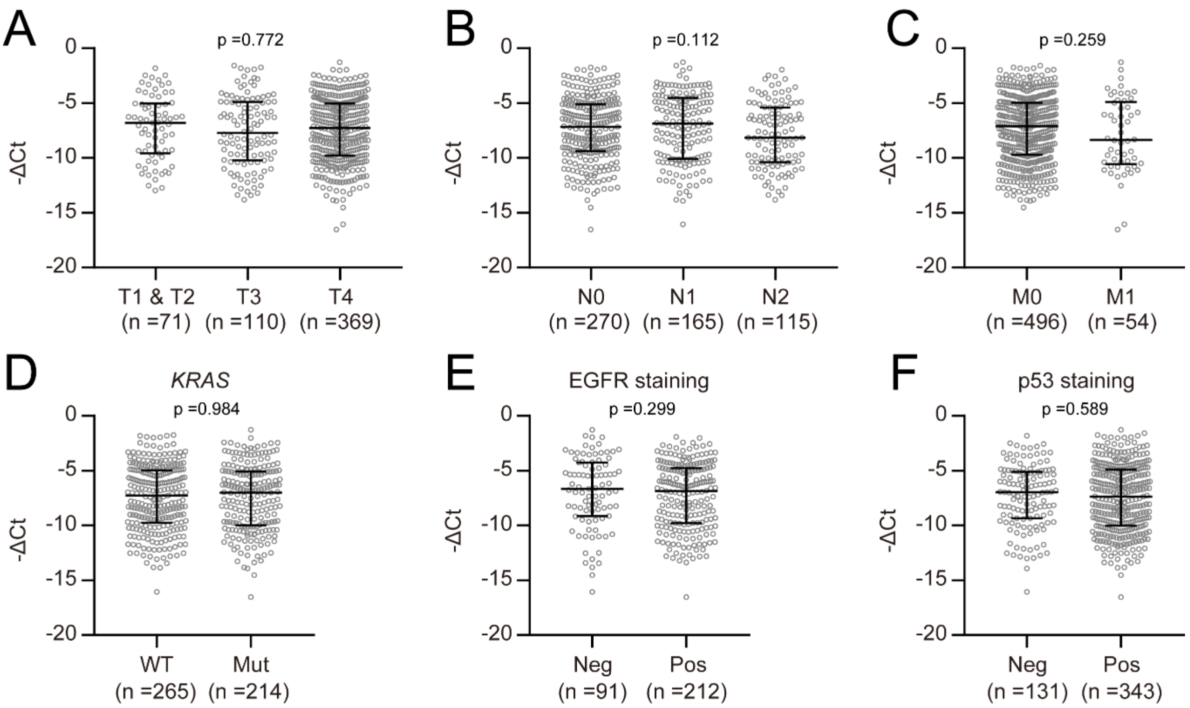


**Figure S8. Relative abundance of *Fusobacterium* in tumour tissues did not vary with any of the examined pathological features of CRC.** (A), (B) and (C) Comparison by T, N, and M stages, respectively. (D) Comparison by *KRAS* mutation status. WT, wild type; Mut, mutation (E) and (F) Comparison of immunohistochemical staining results for EGFR and p53, respectively. Neg, negative; Pos, positive. For (A) and (B), Kruskal-Wallis test followed by Dunn's multiple comparison test. The p values of Kruskal-Wallis test are shown. For (C)–(F), Mann-Whitney test. Individual data points are shown along with the medians and interquartile ranges. All statistical analyses are two-sided where applicable.

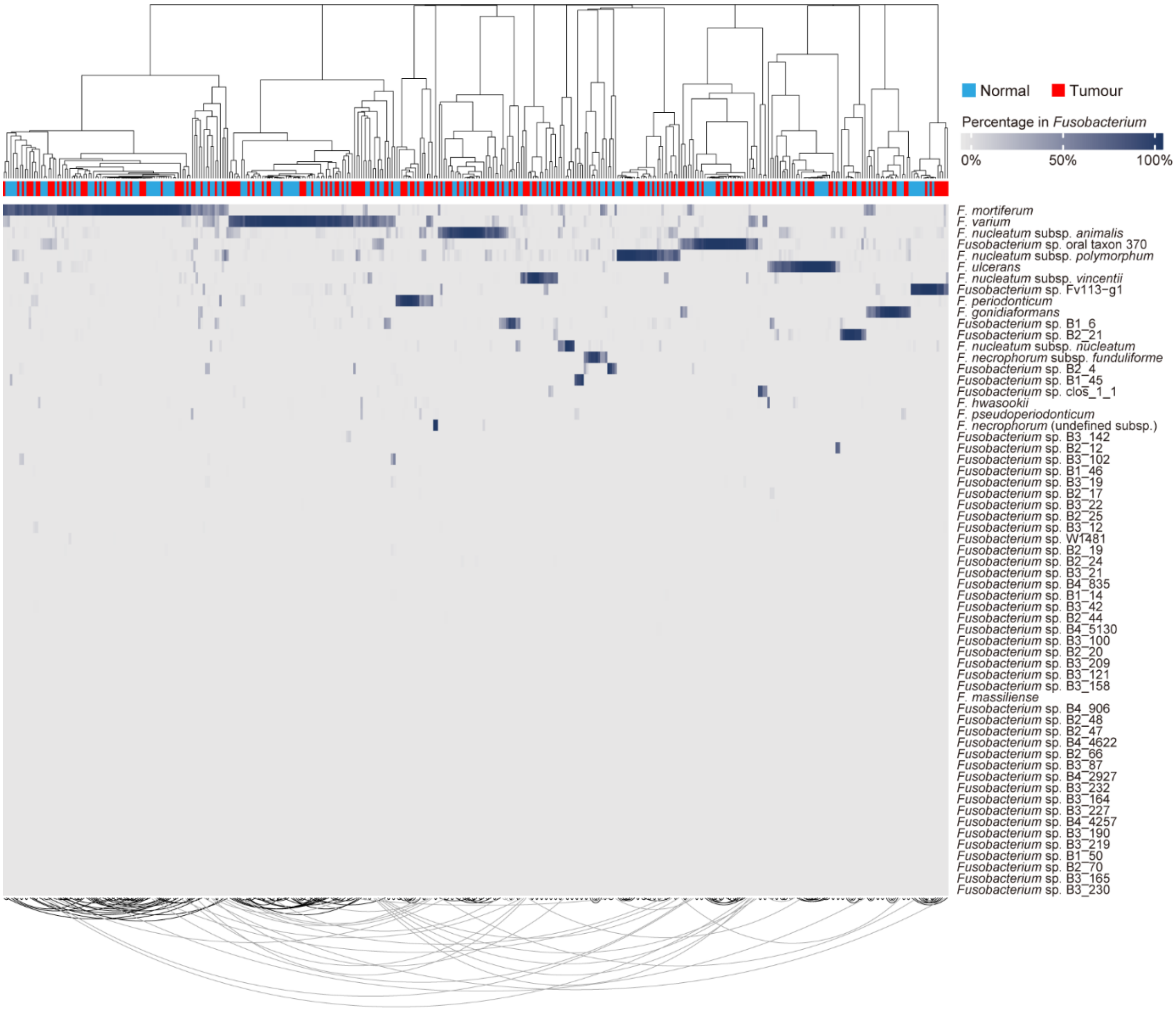


**Figure S9**. ***Fusobacterium* species compositions of 201 paired tumour and normal tissues.** Percentages in the *Fusobacterium* community of each sample are presented as a heatmap. The colour scheme is shown in the figure. Paired samples are connected by lines. The grey lines indicate the paired samples located on separate major branches. The black lines indicate otherwise.

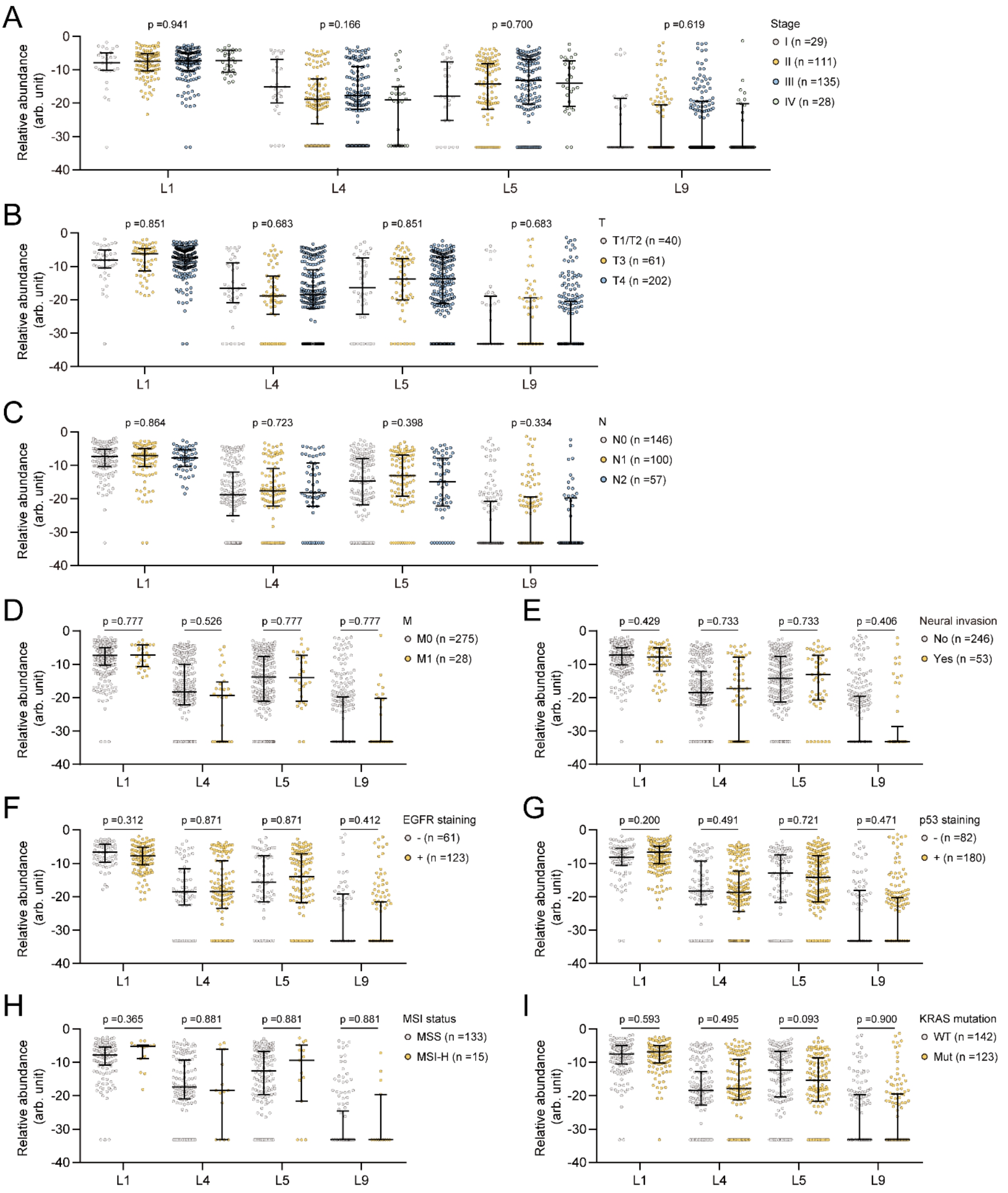


**Figure S10. Associations between lineage abundance and pathological characteristics.** (A) Comparison by stage. (B), (C) and (D) Comparison by tumour, node, and metastasis (TNM) stages, respectively. (E), (F), (G), (H) and (I) Comparison by neural invasion status, EGFR staining result, p53 staining result, MIS status and *KRAS* mutation status, respectively. For (A)-(C), Kruskal-Wallis test followed by Dunn's multiple comparison test was used for each lineage.

Benjamini-Hochberg correction was then applied to correct the p values of the Kruskal-Wallis tests and the adjusted p values are shown for each lineage. For (D)-(I), Mann-Whitney test was used for each lineage and Benjamini-Hochberg correction was then applied. Adjusted p values are shown. Individual data points are shown along with the medians and interquartile ranges. arb. unit, arbitrary unit. All statistical analyses are two-sided where applicable.

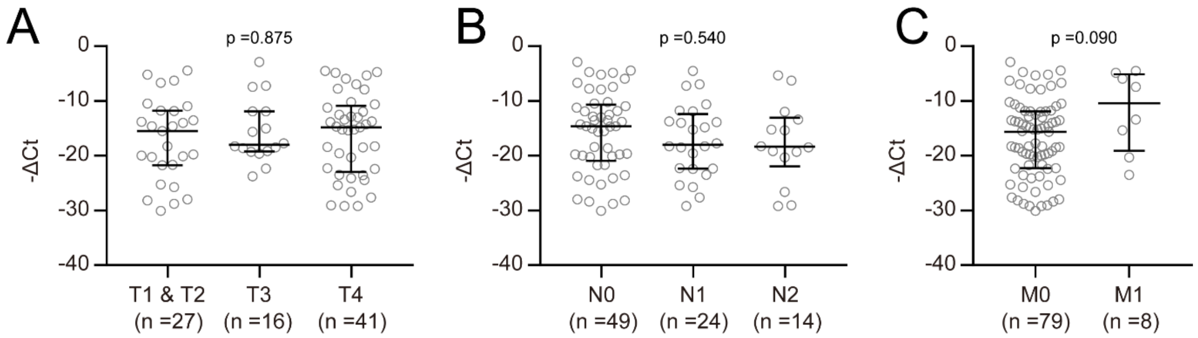


**Figure S11. Relative abundance of *Fusobacterium* in faecal samples from CRC patients compared by tumour, node, metastasis (TNM) stage.** (A), (B) and (C) Comparison by T, N, and M stages, respectively. For (A) and (B), Kruskal-Wallis test followed by Dunn's multiple comparison test. The p values of Kruskal-Wallis test are shown. For (C), Mann-Whitney test. Individual data points are shown along with the medians and interquartile ranges. All statistical analyses are two-sided where applicable.

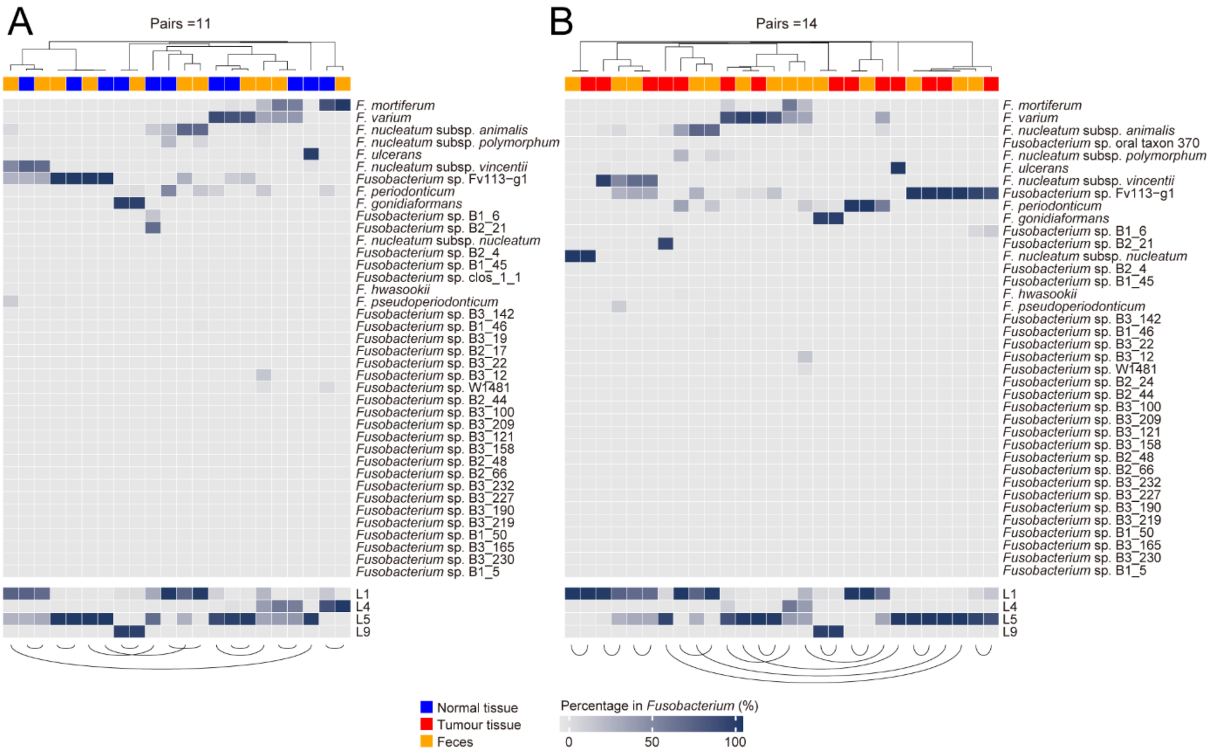


**Figure S12**. ***Fusobacterium* species compositions in faecal samples and matched normal or tumour tissues.** (A) Results for patients with available FrpoB-seq data for faecal samples and matched normal tissues. (B) Results for patients with available FrpoB-seq data for faecal samples and matched tumour tissues. Percentages of specific species within the *Fusobacterium* community of each sample are presented as a heatmap. Paired samples are connected by lines.

Table S1. Bacterial strains used in this study.

| Species | Strain | Reference |
|---|---|---|
| *F. nucleatum* subsp. *nucleatum* | ATCC 25586 | ATCC |
| *F. nucleatum* subsp. *animalis* | THCT5A4 (CCTCC M 2019366) | This study |
| | THCT6B3 (CCTCC M 2019367) | This study |
| | THCT7A2 (CCTCC M 2019365) | This study |
| | THCT5A5 | This study |
| *F. nucleatum* subsp. *polymorphum* | THCT7E2 (CCTCC M 2019364) | This study |
| | THCT15E1 (CCTCC M 2019362) | This study |
| *F. nucleatum* subsp. *vincentii* | THCT14A3 (CCTCC M 2019363) | This study |
| | THCT14B3 | |
| *F. hwasookii* | THCT14E2 (CCTCC M 2019361) | This study |
| *F. varium* | THCT1E1 | This study |
| | THCT1E2 | This study |
| | THCT4E2 | This study |
| | THCT4E4 | This study |
| | THCT13E1 | This study |
| | THCT23E1 | This study |
| | THCT23B1 | This study |
| | THCT23E3 | This study |
| *F. mortiferum* | THCT6B2 | This study |
| *F. pseudoperiodonticum* | THCT18E1 | This study |
| *F. ulcerans* | ATCC 49185 | ATCC |

Table S2. Putative non-specific amplification with the designed universal primers

| Species of hit | Habitat/known origin of isolation |
|---|---|
| *Leptotrichia buccalis* (Fusobacteriales) | human oral and vaginal cavities |
| *Leptotrichia* sp. oral taxon 847 (Fusobacteriales) | human oral cavity |
| *Leptotrichia goodfellowii* (Fusobacteriales) | human faeces and oral and intestinal flora |
| *Sebaldella termitidis* (Fusobacteriales) | termite intestine |
| *Sneathia amnii* (Fusobacteriales) | pathogen of the female urogenital tract |
| *Caviibacter abscessus*/*Streptobacillus moniliformis* (Fusobacteriales) | guinea pigs |
| *Alkaliflexus imshenetskii* | soda lake |
| *Psychrilyobacter atlanticu* (Fusobacteriales) | marine environments |
| *Sneathia sanguinegens* (Fusobacteriales) | human oral cavity and urogenital tract |
| *Streptobacillus notomytis* (Fusobacteriales) | rat (unusual in human) |
| *Leptotrichia trevisanii* (Fusobacteriales) | NA |
| *Labilibacter marinus* | marine sediments |
| *Photobacterium damselae* | marine animals |
| *Alkalitalea saponilacus* | Soap Lake |
| *Clostridium oryzae* | soil |
| *Enterococcus hirae* | zoonotic pathogen (unusual in human) |
| *Bacillus mycoides* | soil |
| *Roseovarius mucosus* | diatom |
| *Bizionia argentinensis* | marine environments |
| *Mycoplasma hyosynoviae* | pig |

Species belonging to the order Fusobacteriales are denoted in parentheses. Information of habitat/known origin of isolation was retrieve from the NCBI database. NA, not available.

Table S3. Annotation of non-specific sequences obtained by FrpoB-seq

| Species in which the non-specific sequences were found or had the best hits |
|---|
| *Homo sapiens* |
| *Leptotrichia buccalis* |
| *Leptotrichia hongkongensis* |
| *Leptotrichia trevisanii* |
| *Leptotrichia wadei* |
| *Leptotrichia sp. oral taxon 498* |
| *Leptotrichia sp. oral taxon 212* |
| *Clostridium* |
| *Eubacterium* |
| *Akkermansia muciniphila* |
| *Alistipes* |
| *Anaerostipes hadrus* |
| *Aphantopus hyperantus* |
| *Arabia massiliensis* |
| *Bacteroides* |
| *Blautia* |
| *Burkholderiales* |
| *Butyricimonas faecalis* |
| *Coprococcus catus* |
| *Danio kyathit* |
| *Desulfovibrio fairfieldensis* |
| *Dysosmobacter welbionis* |
| *Eikenella corrodens* |
| *Enterocloster clostridioformis* |
| *Erithacus rubecula* |
| *Erysipelatoclostridium ramosum* |
| *Escherichia coli* |
| *Faecalibacterium prausnitzii* |
| *Flavonifractor plautii* |
| *Lachnospiraceae* |
| *Lactobacillus rennini* |
| *Megamonas funiformis* |
| *Parabacteroides* distasonis |
| *Paraprevotella xylaniphila* |
| *Phocaeicola* |
| *Poecilia reticulate* |
| *Porphyromonas crevioricanis* |
| *Prevotella* |
| *Roseburia intestinalis* |
| *Selenomonas sp. oral taxon 136* |
| *Spirometra erinaceieuropaei* |

| |
|---|
| *Streptococcus* |
| *Veillonella* |
| *Victivallales* |
| Unknown |
