# Supplementary material for: Profiling Fusobacterium infection at high taxonomic resolution reveals lineage-specific correlations in colorectal cancer
Source: Nat Commun. 2022 Jun 9;13:3336. doi: 10.1038/s41467-022-30957-6 (PMC9184491; doi:10.1038/s41467-022-30957-6)
Supplement: Supplementary file 2 — Description of Additional Supplementary Files [file 41467_2022_30957_MOESM2_ESM.pdf]

Description of Additional Supplementary Files

File Name: Supplementary Data 1
Description: Bacterial genomes used in this study.

File Name: Supplementary Data 2
Description: ANIb values calculated in pairwise.

File Name: Supplementary Data 3
Description: FrpoB-seq results in the tissue and faecal samples.

File Name: Supplementary Data 4
Description: Pathological information of the patients with FrpoB-seq data available in tumour tissues.

File Name: Supplementary Data 5
Description: Pathological information of the patients and characteristics of health volunteers with FrpoB-seq data available in faecal samples.
