# Supplementary material for: Profiling Fusobacterium infection at high taxonomic resolution reveals lineage-specific correlations in colorectal cancer
Source: Nat Commun. 2022 Jun 9;13:3336. doi: 10.1038/s41467-022-30957-6 (PMC9184491; doi:10.1038/s41467-022-30957-6)
Supplement: Supplementary file 8 — Reporting Summary [file 41467_2022_30957_MOESM8_ESM.pdf]

nature portfolio

Corresponding author(s): Qing Wei and Huanlong Qin

Last updated by author(s): May 5, 2022

Reporting Summary

Nature Portfolio wishes to improve the reproducibility of the work that we publish. This form provides structure for consistency and transparency in reporting. For further information on Nature Portfolio policies, see our Editorial Policies and the Editorial Policy Checklist.

Statistics

For all statistical analyses, confirm that the following items are present in the figure legend, table legend, main text, or Methods section.

n/a	Confirmed	
☐	☒	The exact sample size (*n*) for each experimental group/condition, given as a discrete number and unit of measurement
☐	☒	A statement on whether measurements were taken from distinct samples or whether the same sample was measured repeatedly
☐	☒	The statistical test(s) used AND whether they are one- or two-sided<br>*Only common tests should be described solely by name; describe more complex techniques in the Methods section.*
☐	☒	A description of all covariates tested
☐	☒	A description of any assumptions or corrections, such as tests of normality and adjustment for multiple comparisons
☐	☒	A full description of the statistical parameters including central tendency (e.g. means) or other basic estimates (e.g. regression coefficient) AND variation (e.g. standard deviation) or associated estimates of uncertainty (e.g. confidence intervals)
☐	☒	For null hypothesis testing, the test statistic (e.g. *F*, *t*, *r*) with confidence intervals, effect sizes, degrees of freedom and *P* value noted<br>*Give P values as exact values whenever suitable.*
☒	☐	For Bayesian analysis, information on the choice of priors and Markov chain Monte Carlo settings
☒	☐	For hierarchical and complex designs, identification of the appropriate level for tests and full reporting of outcomes
☐	☒	Estimates of effect sizes (e.g. Cohen's *d*, Pearson's *r*), indicating how they were calculated

Our web collection on statistics for biologists contains articles on many of the points above.

Software and code

Policy information about availability of computer code

Data collection	The SRA Toolkit 2.11.2 was used to download metagenomic data from NCBI.
Data analysis	For bacterial genome sequencing data, PBdagcon (https://github.com/PacificBiosciences/pbdagcon) was used for subread correction. GATK v1.6 (https://github.com/broadinstitute/gatk) and the SOAP tool package were used for single-base correction; genomes were assembled with Celera Assembler v8.3 (http://wgs-assembler.sourceforge.net); coding genes were predicted with Glimmer v3.02, while non-coding genes were annotated with RNAmmer v1.2 and tRNAscan-SE v1.31. Pairwise whole-genome average nucleotide identity calculated with BLAST (ANIb) analysis was conducted with JSpecies v1.2.1. Multiple sequence alignments were performed with MUSCLE, and phylogenetic trees were subsequently constructed with MEGA5 30 using the maximum likelihood algorithm and 1,000 bootstrap replicates. Whole genome-based phylogenetic analysis was conducted with kSNP3. For FrpoB-seq data, paired-end reads with an overlap of ≥15 bp and a mismatch rate of <0.1 were assembled with FLASH v1.2.11 and operational taxonomic units (OTUs) were generated with 100% identity via USEARCH (https://drive5.com/usearch/). For metagenomic sequencing data, Contigs were assembled with MEGAHIT. Heatmaps were generated with the gplots (https://github.com/talgalili/gplots) or ComplexHeatmap package in R (version 4.0.2).. Statistical analyses were performed with GraphPad Prism (version 5), SPSS (version 19) or R (version 4.0.2).

For manuscripts utilizing custom algorithms or software that are central to the research but not yet described in published literature, software must be made available to editors and reviewers. We strongly encourage code deposition in a community repository (e.g. GitHub). See the Nature Portfolio guidelines for submitting code & software for further information.

Data

Policy information about availability of data

All manuscripts must include a data availability statement. This statement should provide the following information, where applicable:

- Accession codes, unique identifiers, or web links for publicly available datasets
- A description of any restrictions on data availability
- For clinical datasets or third party data, please ensure that the statement adheres to our policy

The data generated or analysed during this study are included within the paper, its Supplementary Information/Data files, and public repositories. The bacterial genome data generated in this study have been deposited in the NCBI GenBank database under the accession CP071099 [https://www.ncbi.nlm.nih.gov/nuccore/CP071099] for strain THCT5A4, CP071098 [https://www.ncbi.nlm.nih.gov/nuccore/CP071098] for THCT6B3, CP071097 [https://www.ncbi.nlm.nih.gov/nuccore/CP071097] for THCT7A2, CP071096 [https://www.ncbi.nlm.nih.gov/nuccore/CP071096] for THCT7E2, CP071093 [https://www.ncbi.nlm.nih.gov/nuccore/CP071093] for THCT14A3, CP071092 [https://www.ncbi.nlm.nih.gov/nuccore/CP071092] for THCT14E2 and CP071094 [https://www.ncbi.nlm.nih.gov/nuccore/CP071094] - CP071095 [https://www.ncbi.nlm.nih.gov/nuccore/CP071095] for THCT15E1. Other raw sequencing data generated in this study have been deposited in the NCBI SRA database under the accession number PRJNA715828 [https://www.ncbi.nlm.nih.gov/sra/?term=PRJNA715828]. Detailed information of 192 publicly available Fusobacterium genomes retrieved from the NCBI nucleotide database is listed in Dataset S1 with accession numbers included. The publicly available metagenomic datasets used were retrieved from the NCBI SRA database under the accessions PRJNA557323 [https://www.ncbi.nlm.nih.gov/sra/?term=PRJNA557323] and PRJNA678426 [https://www.ncbi.nlm.nih.gov/sra/?term=PRJNA678426]. Source data are provided with this paper.

Field-specific reporting

Please select the one below that is the best fit for your research. If you are not sure, read the appropriate sections before making your selection.

☒ Life sciences ☐ Behavioural & social sciences ☐ Ecological, evolutionary & environmental sciences

For a reference copy of the document with all sections, see nature.com/documents/nr-reporting-summary-flat.pdf

Life sciences study design

All studies must disclose on these points even when the disclosure is negative.

Sample size	The sample size were chosen based on studying previous researches, including Guinney, et al. (Nat Med, 2015) and Yeoh, et al. (Gut, 2020).
Data exclusions	All generated data were included for analysis.
Replication	For large-scale qPCR, an independent repeat was made for each sample and mean value was used. For tests having a SD >0.5 (Ct value), measurement with two repeats was performed again. For standard PCR, experiment was performed in triplicate. Due to resource limitation, FrpoB-seq was not repeated for each sample except the mock sample, which was independently tested three times. Genome sequencing of bacterial strains and metagenomic sequencing was conducted once for each relevant sample.
Randomization	This is a retrospective observational study. Randomization is not applicable
Blinding	Investigators who conducted qPCR measurement, standard PCR and FrpoB-seq were blind to the sample groups. Blinding was not relevant to to other experiment or data collection/analysis as in those situations most of the results were machine/sofeware-generated based on the qPCR and FrpoB-seq data or data directly retrieved from the public database, blinding or not would unlikely affect the process and the relevant results. In addition, sometimes, the investigators may also need to choose corresponding methods (e.g. statistical test) based on the data.

Reporting for specific materials, systems and methods

We require information from authors about some types of materials, experimental systems and methods used in many studies. Here, indicate whether each material, system or method listed is relevant to your study. If you are not sure if a list item applies to your research, read the appropriate section before selecting a response.

Materials & experimental systems

n/a	Involved in the study	
☒	☐	Antibodies
☒	☐	Eukaryotic cell lines
☒	☐	Palaeontology and archaeology
☒	☐	Animals and other organisms
☐	☒	Human research participants
☒	☐	Clinical data
☒	☐	Dual use research of concern

Methods

n/a	Involved in the study	
☒	☐	ChIP-seq
☒	☐	Flow cytometry
☒	☐	MRI-based neuroimaging

Human research participants

Policy information about studies involving human research participants

Population characteristics	Provided in supplementary datasets (Datasets S4 and S5).
Recruitment	Specimens of patients were retrieved from the Biobank of Shanghai Tenth People's Hospital. Heath volunteers were recruited in the same centre from those who underwent health examination. It should be noted that there may be potential selection bias for the health volunteers. The volunteers who underwent health examination may have awareness of keeping good life styles thus may not sufficiently represent the entire heath population. Also, this is a single-centre study and the sample size of health volunteers is relatively small. Future validation is needed.
Ethics oversight	Ethics Committee of Shanghai Tenth People’s Hospital

Note that full information on the approval of the study protocol must also be provided in the manuscript.
